# An inventory of human light exposure behaviour

**DOI:** 10.1038/s41598-023-48241-y

**Published:** 2023-12-13

**Authors:** Mushfiqul Anwar Siraji, Rafael Robert Lazar, Juliëtte van Duijnhoven, Luc J. M. Schlangen, Shamsul Haque, Vineetha Kalavally, Céline Vetter, Gena L. Glickman, Karin C. H. J. Smolders, Manuel Spitschan

**Affiliations:** 1 Department of Psychology, Jeffrey Cheah School of Medicine and Health Sciences, Monash University Malaysia, Selangor, Malaysia; 2 Department of History and Philosophy, North South University, Dhaka, Bangladesh; 3 Centre for Chronobiology, Psychiatric Hospital of the University of Basel (UPK), Basel, Switzerland; 4 Research Cluster Molecular and Cognitive Neurosciences, University of Basel, Basel, Switzerland; 5 Department of the Built Environment, Building Lighting, Eindhoven University of Technology, Eindhoven, The Netherlands; 6 Intelligent Lighting Institute, Eindhoven University of Technology, Eindhoven, The Netherlands; 7 Department of Industrial Engineering and Innovation Sciences, Human-Technology Interaction, Eindhoven University of Technology, Eindhoven, The Netherlands; 8 Department of Electrical and Computer Systems Engineering, Monash University Malaysia, Selangor, Malaysia; 9 Department of Integrative Physiology, University of Colorado Boulder, Boulder, USA; 10 Present Address: IQVIA GmbH, Frankfurt am Main, Germany; 11 Department of Psychiatry, Uniformed Services University of the Health Sciences, Bethesda, USA; 12 Translational Sensory & Circadian Neuroscience, Max Planck Institute for Biological Cybernetics, Tübingen, Germany; 13 TUM School of Medicine and Health, Technical University of Munich, Munich, Germany; 14 TUM Institute of Advanced Study (TUM-IAS), Technical University of Munich, Garching, Germany

**Keywords:** Psychology, Human behaviour

## Abstract

Light exposure is an essential driver of health and well-being, and individual behaviours during rest and activity modulate physiologically relevant aspects of light exposure. Further understanding the behaviours that influence individual photic exposure patterns may provide insight into the volitional contributions to the physiological effects of light and guide behavioural points of intervention. Here, we present a novel, self-reported and psychometrically validated inventory to capture light exposure-related behaviour, the Light Exposure Behaviour Assessment (LEBA). An expert panel prepared the initial 48-item pool spanning different light exposure-related behaviours. Responses, consisting of rating the frequency of engaging in the per-item behaviour on a five-point Likert-type scale, were collected in an online survey yielding responses from a geographically unconstrained sample (690 completed responses, 74 countries, 28 time zones). The exploratory factor analysis (EFA) on an initial subsample (n = 428) rendered a five-factor solution with 25 items (wearing blue light filters, spending time outdoors, using a phone and smartwatch in bed, using light before bedtime, using light in the morning and during daytime). In a confirmatory factor analysis (CFA) performed on an independent subset of participants (n = 262), we removed two additional items to attain the best fit for the five-factor solution (CFI = 0.95, TLI = 0.95, RMSEA = 0.06). The internal consistency reliability coefficient for the total instrument yielded McDonald’s Omega = 0.68. Measurement model invariance analysis between native and non-native English speakers showed our model attained the highest level of invariance (residual invariance CFI = 0.95, TLI = 0.95, RMSEA = 0.05). Lastly, a short form of the LEBA (n = 18 items) was developed using Item Response Theory on the complete sample (n = 690). The psychometric properties of the LEBA indicate the usability for measuring light exposure-related behaviours. The instrument may offer a scalable solution to characterise behaviours that influence individual photic exposure patterns in remote samples. The LEBA inventory is available under the open-access CC-BY license. Instrument webpage: https://leba-instrument.org/ GitHub repository containing this manuscript: https://github.com/leba-instrument/leba-manuscript.

## Introduction

Light exposure received by the eyes affects many facets of human health, well-being, and performance beyond visual sensation and perception^1^. The non-image-forming (NIF) effects of light comprise light’s circadian and non-circadian influence on several physiological and psychological functions, such as the secretion of melatonin, sleep, mood, pupil size, body temperature, alertness, and higher cognitive functions^2^.

With the introduction of artificial electric light, human behaviour has become dissociated from the light-dark cycle given by solar radiation. People can now frequently choose when to be exposed to light or darkness. For example, they can decide whether to go outdoors and seek out sunlight, switch on/off light-emitting devices, use certain types of lights at home, or avoid specific light environments altogether. Additionally, when light sources cannot be directly manipulated, sought out, or avoided (for example, at school, work, or in public places), there is still potential leeway to influence personal light exposure behaviourally, for instance, by wearing sunglasses, directing one’s gaze away or supplementing the situation with additional light sources. Although clearly yielding the potential for good, these behaviours are further associated with increased electric light exposure at night and indoor time during the day, compromising the natural temporal organisation of the light-dark cycle. For example, in the US, an average of 87% of the time is spent in enclosed buildings^3^, and more than 80% of the population is exposed to a night sky that is brighter than nights with a full moon due to electric light at night^4^.

An extensive body of scientific evidence suggests that improper light exposure may be disruptive to health and well-being, giving rise to a series of adverse consequences, including the alteration of hormonal rhythms, increased cancer rates, cardiovascular diseases, and metabolic disorders, such as obesity and type II diabetes^4–6^. These findings have sparked a significant call for assessment and guidance regarding healthy light exposure, as exemplified by a recently published set of consensus-based experts’ recommendations with specific requirements for indoor light environments during the daytime, evening, and nighttime^7^.

Furthermore, building on earlier attempts^8^, there was a recent push toward the development and use of portable light loggers to improve ambulant light assessment and gain more insight into the NIF effects of light on human health in field conditions^9,10^. Attached to different body parts (e.g., wrist, head, at eye level, chest), these light loggers allow for the objective measurement of individual photic exposure patterns under real-world conditions and thus are valuable tools for field studies. Nevertheless, these devices also encompass limiting factors such as potentially being intrusive (e.g., when eye-level worn), yielding the risk of getting covered (e.g., when wrist- or chest-worn) and requiring (monetary) resources and expertise for acquisition and maintenance of the devices. Moreover, it is important to note that portable light loggers alone do not collect data on the specific behavioural patterns in relation to light exposure.

On the other hand, several attempts have been made to quantify received light exposure subjectively with self-report questionnaires^11–20^ (see Supplementary Table 1). However, self-reporting light properties could be challenging for people who lack technical knowledge of light sources. Moreover, it is worth considering that the human visual system, unlike a photometer, continuously adapts to ambient brightness^21^, while the signals underlying the non-visual effects of light are independent from perception^22^. Retrospectively recalling the properties of a light source can further complicate such subjective evaluations. Moreover, measuring light properties alone does not yield any information about how individuals might behave differently regarding diverse light environments such as work, at home or outdoors.

To date, little effort has been made to understand and capture these activities. Here, we present the development process of a novel self-reported inventory, the Light Exposure Behaviour Assessment (LEBA), for characterising diverse light exposure-related behaviours. Notably, the focus of the LEBA inventory is not to estimate personal light exposure. Instead, we aim to assess, in a scalable way, how people behave in relation to light, focusing on habitual patterns that could guide behavioural interventions.

**Table 1:** Demographic characteristics of participants (n = 690).

Variable	Overall, N = 690^1^	1. EFA Sample, N = 428^1^	2. CFA Sample, N = 262^1^
Age	32.95 (14.57)	32.99 (15.11)	32.89 (13.66)
Sex			
Female	325 (47%)	189 (44%)	136 (52%)
Male	351 (51%)	230 (54%)	121 (46%)
Other	14 (2%)	9 (2%)	5 (2%)
Gender-Variant Identity	49 (7%)	33 (8%)	16 (6%)
Native English Speaker	320 (46%)	191 (45%)	129 (49%)
Occupational Status			
Work	396 (57%)	235 (55%)	161 (61%)
School	174 (25%)	122 (29%)	52 (20%)
Neither	120 (17%)	71 (17%)	49 (19%)
Occupational setting			
Home office/Home schooling	303 (44%)	194 (45%)	109 (42%)
Face-to-face work/Face-to-face schooling	109 (16%)	68 (16%)	41 (16%)
Combination of home- and face-to-face- work/schooling	147 (21%)	94 (22%)	53 (20%)
Neither (no work or school, or in vacation)	131 (19%)	72 (17%)	59 (23%)

^1^ Mean (SD); n (%).

## Results

Our results focus on the development of the LEBA inventory and its psychometric validation using a large-scale online sample dataset (n = 690).

### Development of the initial item pool

To capture the human light exposure-related behaviours, 48 items were developed by an expert panel (all authors—researchers from chronobiology, light research, neuroscience and psychology in different geographical contexts). Face validity examination by each panel member indicated all items were relevant, and a few modifications were suggested. The author team discussed the suggestions and amended the items as indicated, thus creating a 48-item inventory.

### Measurement of light exposure behaviour in an online sample

We conducted two rounds of a large-scale online survey between 17 May 2021 and 3 September 2021 to generate data from 690 participants with varied geographic locations (countries = 74; time-zone = 28). For a complete list of geographic locations, see Supplementary Table 2. Table 1 presents the survey participants’ demographic characteristics. Only participants completing the full LEBA inventory were included. We used the data from the first round for the exploratory factor analysis (EFA sample; n = 428) and data from the second round for the confirmatory factor analysis (CFA sample; n = 262). Participants in our survey were aged between 11 to 84 years, with an overall mean of ~ 32.95 years of age [Overall: 32.95 ± 14.57; EFA: 32.99 ± 15.11; CFA: 32.89 ± 13.66]. In the entire sample, 351 (51%) were male, 325 (47%) were female, 14 (2%) reported other sex, and 49 (7%) reported a gender-variant identity. In a “Yes/No” question regarding native language, 320 (46%) of respondents [EFA: 191 (45%); CFA: 129 (49%)] indicated that they were native English speakers. For their “Occupational Status”, more than half of the overall sample (396 (57%)) reported that they currently work, whereas 174 (25%) reported that they go to school, and 120 (17%) responded that they do “Neither”. With respect to the COVID-19 pandemic, we asked participants to indicate their occupational setting during the last four weeks: In the entire sample, 303 (44%) of the participants indicated that they were in a home office/ home schooling setting, 109 (16%) reported face-to-face work/schooling, 147 (21%) reported a combination of home- and face-to-face work/schooling, and 131 (19%) filled in the “Neither (no work or school, or on vacation)” response option.

### Psychometric analysis: development of the long form

#### Descriptive statistics and item analysis

**Figure 1:**
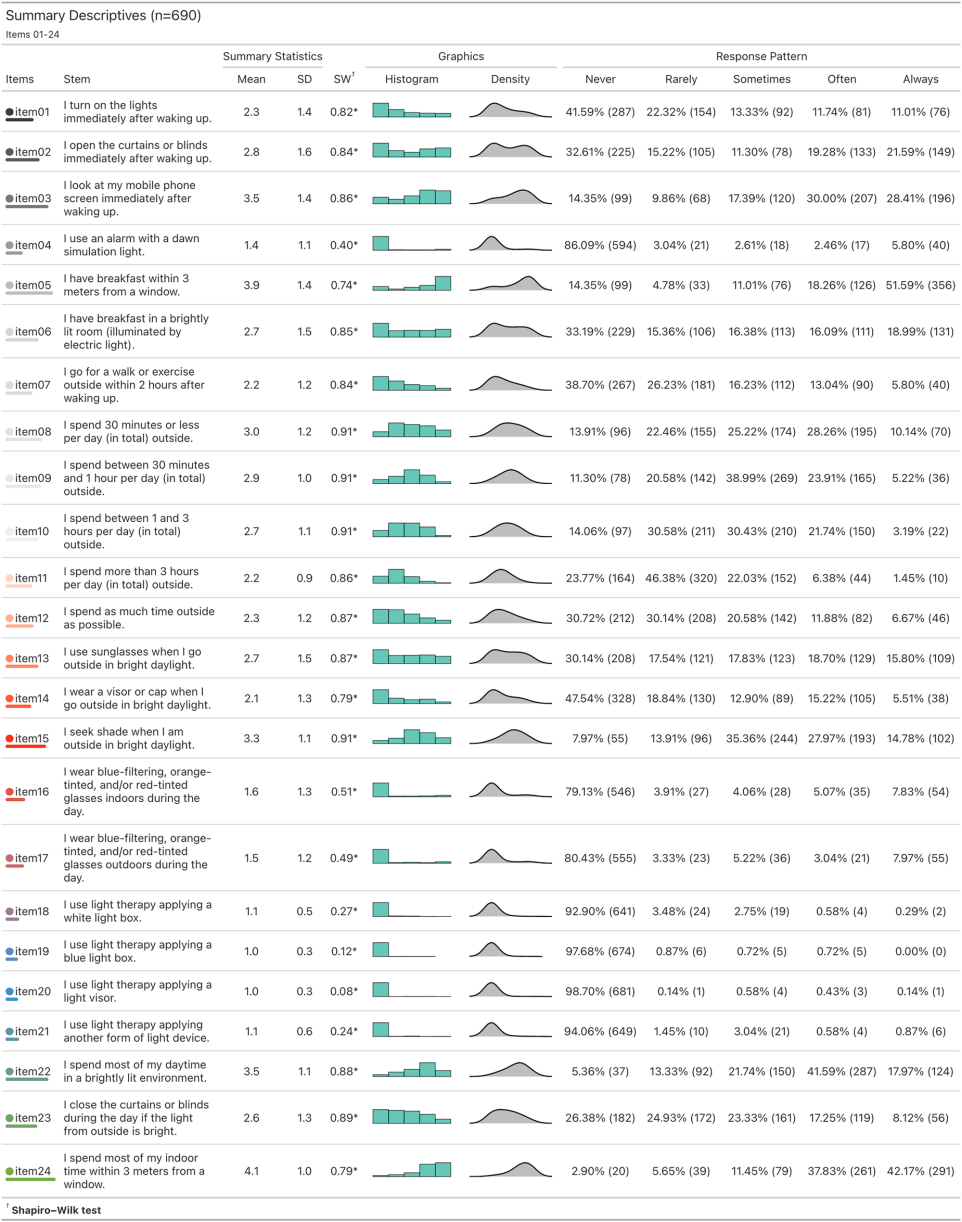
Summary descriptives and response patterns observed in the large-scale survey for items 01-24. ‘*’ denotes a significant deviation from the normality assumption according to the Shapiro-Wilk test. All items violated the normality assumption.

**Figure 2:**
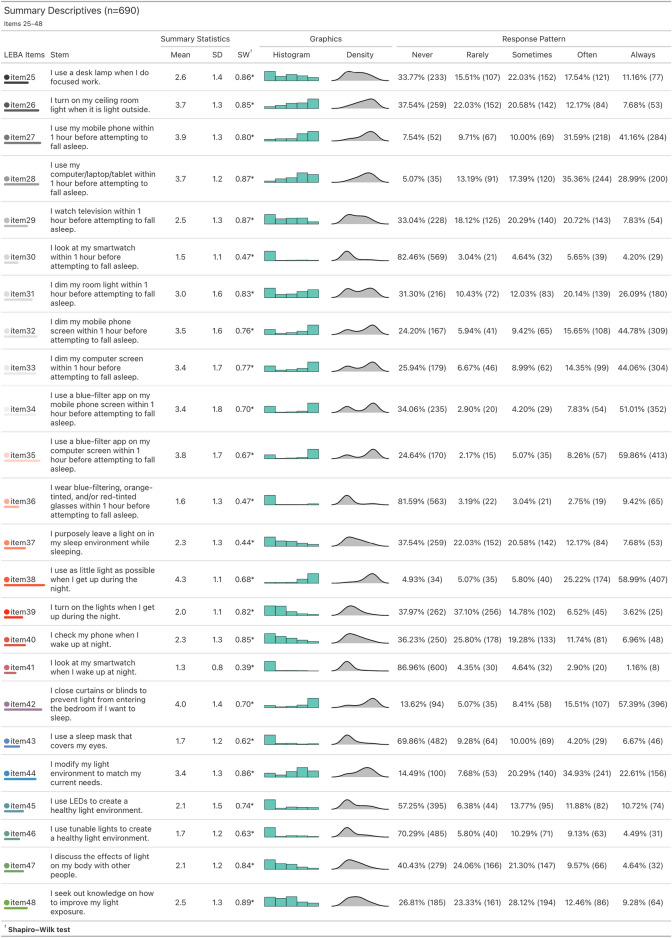
Summary descriptives and response patterns observed in the large-scale survey for items 25–48. ‘*’ denotes a significant deviation from the normality assumption according to the Shapiro-Wilk test. All items violated the normality assumption.

We observed that the response patterns of the LEBA inventory for the entire sample (n = 690) were not normally distributed (Figs. 1 and 2). All items violated both univariate^23^ and multivariate normality^24^. The multivariate skewness was 488.40 (p < 0.001), and the multivariate kurtosis was 2808.17 (p < 0.001).

Similarly, a non-normal distribution of response patterns was also observed in the EFA sample. Supplementary Figure 1 depicts the univariate descriptive statistics for the EFA sample (n = 428). Further, we observed that each item’s correlation with the aggregated sum of the 48-item score varied largely (corrected item-total correlation = 0.03–0.48), indicating the possibility of a multi-factor structure of the LEBA inventory.

#### Exploratory factor analysis and reliability analysis

Exploratory analysis revealed that items of LEBA inventory could be categorised into five major factors: (i) wearing blue light filters; (ii) spending time outdoors; (iii) using a phone and smartwatch in bed; (iv) using light before bedtime (v) using light in the morning and during daytime. In this stage of analysis, we retained 25 items. The first factor had three items and encapsulated the individual’s preference for using blue light filters in different light environments. The second factor contained six items that incorporated the individuals’ hours spent outdoors. The third factor contained five items that looked into specific behaviours of using a phone and smartwatch in bed. The fourth factor comprised five items that investigated other behaviours related to the individual’s exposure to electric light before bedtime. Lastly, the fifth factor encompassed six items capturing the individual’s morning and daytime light exposure-related behaviour.

**Figure 3:**
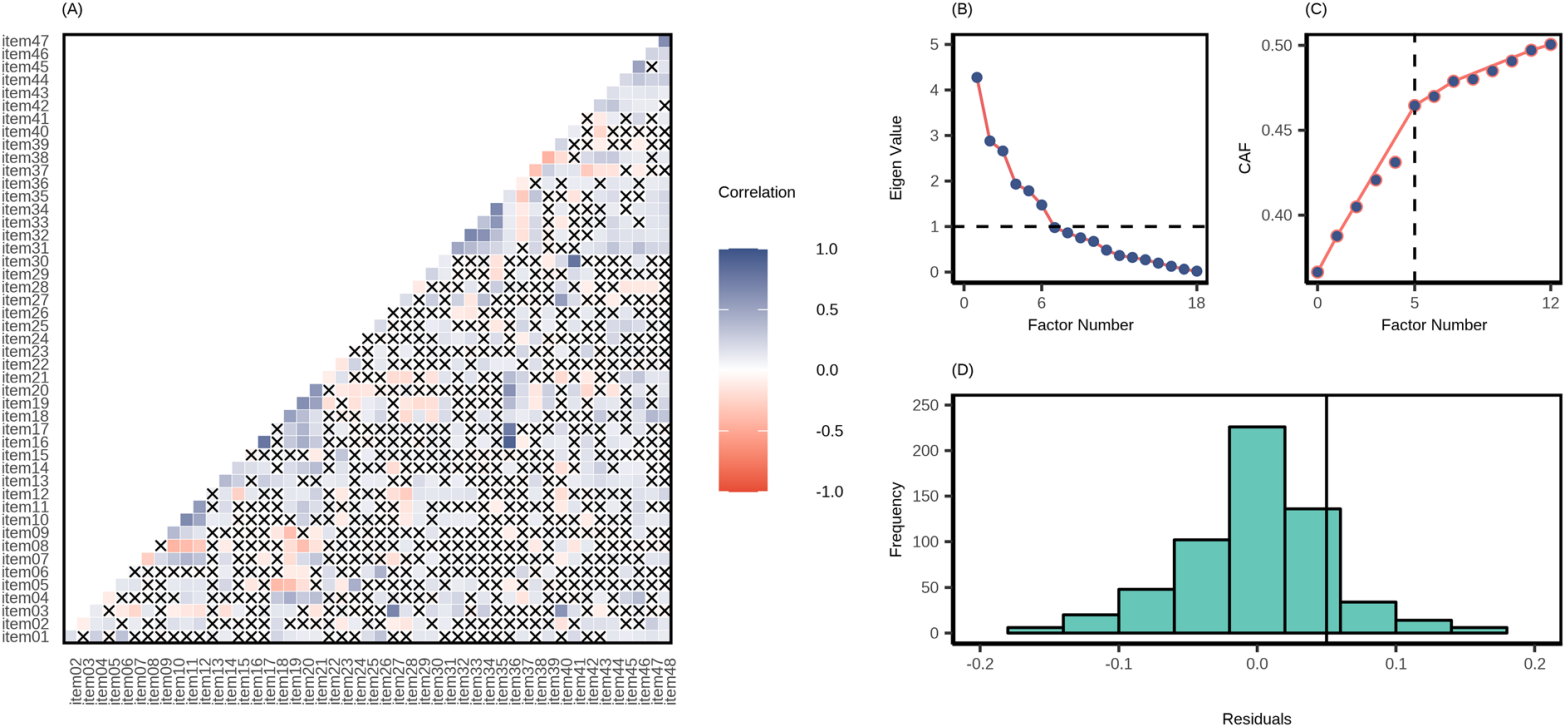
(**A**) Inter-item polychoric correlation coefficients for the 48 items. 4.9% of inter-item correlation coefficients were higher than |0.3|. ‘x’ denotes a non-significant correlation. (**B**) The Scree plot suggested six factors. (**C**) Hull method indicated that five factors were required to balance the model fit and number of parameters. (**D**) The histogram of nonredundant residual correlations in the five-factor model indicated that 26% of inter-item correlations were higher than 0.05, hinting at a possible under-factoring.

Prior to conducting the EFA, we checked the post-hoc sampling adequacy by applying Kaiser-Meyer-Olkin (KMO) measures of sampling adequacy on the EFA sample (n = 428)^25^ and the quality of the correlation matrix by Bartlett’s test of sphericity^26^. KMO >0.5 would indicate adequate sample size^27^, and a significant test of sphericity would indicate satisfactory quality of the correlation matrix. Results indicated that we had an adequate sample size (KMO = 0.63) and correlation matrix ($\chi ^2_{1128}$ = 5042.86, p < 0.001). However, 4.96% of the inter-item correlation coefficients were greater than |0.30|, and the inter-item correlation coefficients ranged between − 0.44 to 0.91. Figure 3A depicts the respective correlation matrix. To identify how many factors are required to optimally express human light exposure-related behaviours, we used a combination of methods. The Scree plot (Fig. 3B) revealed a six-factor solution, whereas the minimum average partial (MAP) method^28^ (Supplementary Table 3) and Hull method^29^ implied a five-factor solution (Fig. 3C). Hence, we tested both five-factor and six-factor solutions using iterative EFA, where we gradually identified and discarded problematic items (factor-loading <0.3 and cross-loading >0.3). In this process, we found a five-factor structure for the LEBA inventory with 25 items. Table 2 displays the factor-loadings ($\lambda$) and communalities of the items. Both factor loadings and communalities advocate accepting this five-factor solution ( |$\lambda$|= 0.32–0.99; commonalities = 0.11–0.99). These five factors explain 10.25%, 9.93%, 8.83%, 8.44%, and 6.14% of the total variance in individuals’ light exposure-related behaviours, respectively. All factors exhibited excellent to satisfactory reliability (ordinal $\alpha$ = 0.94, 0.76, 0.75, 0.72, 0.62, respectively). The entire inventory also exhibited satisfactory reliability ($\omega _t$ = 0.77).

However, the histogram of the absolute values of nonredundant residual correlations (Fig. 3D) displayed that 26% of correlations were greater >|.05|, indicating a possible under-factoring^30^. Subsequently, we fitted a six-factor solution, where a factor with only two salient variables emerged, thus disqualifying the six-factor solution (Supplementary Table 4). While making the judgement of accepting this five-factor solution, we considered both factors’ interpretability and their psychometric properties. We deemed the five derived factors as highly interpretable and relevant concerning our aim to capture light exposure-related behaviour, and we retained all of them with 25 items. Two of the items showed negative factor-loading (item 08: “I spend 30 min or less per day (in total) outside.” and item 37: “I use a blue-filter app on my computer screen within 1 h before attempting to fall asleep.”). Upon re-inspection, we recognised these items to be negatively correlated to the respective factor, and thus, we reverse-scored these two items.

**Table 2:** Factor loadings and communality of the retained items in EFA using principal axis extraction method (n = 482).

Item	Stem	PA1	PA2	PA3	PA4	PA5	Communality
Item16	I wear blue-filtering, orange-tinted, and/or red-tinted glasses indoors during the day.	0.99					0.99
Item36	I wear blue-filtering, orange-tinted, and/or red-tinted glasses within 1 h before attempting to fall asleep.	0.94					0.90
Item17	I wear blue-filtering, orange-tinted, and/or red-tinted glasses outdoors during the day.	0.8					0.66
Item11	I spend more than 3 h per day (in total) outside.		0.79				0.64
Item10	I spend between 1 and 3 h per day (in total) outside.		0.76				0.59
Item12	I spend as much time outside as possible.		0.65				0.47
Item07	I go for a walk or exercise outside within 2 h after waking up.		0.5				0.27
Item08	I spend 30 min or less per day (in total) outside.		− 0.49				0.25
Item09	I spend between 30 min and 1 h per day (in total) outside.		0.32				0.11
Item27	I use my mobile phone within 1 h before attempting to fall asleep.			0.8			0.66
Item03	I look at my mobile phone screen immediately after waking up.			0.8			0.68
Item40	I check my phone when I wake up at night.			0.65			0.46
Item30	I look at my smartwatch within 1 h before attempting to fall asleep.			0.45			0.35
Item41	I look at my smartwatch when I wake up at night.			0.36			0.33
Item33	I dim my computer screen within 1 h before attempting to fall asleep.				0.74		0.56
Item32	I dim my mobile phone screen within 1 h before attempting to fall asleep.				0.73		0.62
Item35	I use a blue-filter app on my computer screen within 1 h before attempting to fall asleep.				0.66		0.45
Item37	I purposely leave a light on in my sleep environment while sleeping.				− 0.39		0.17
Item38	I use as little light as possible when I get up during the night.				0.38		0.18
Item46	I use tunable lights to create a healthy light environment.					0.6	0.42
Item45	I use LEDs to create a healthy light environment.					0.59	0.37
Item25	I use a desk lamp when I do focused work.					0.41	0.19
Item04	I use an alarm with a dawn simulation light.					0.41	0.22
Item01	I turn on the lights immediately after waking up.					0.4	0.17
Item26	I turn on my ceiling room light when it is light outside.					0.35	0.16

*Note* Only loading > 0.3 is reported.

#### Confirmatory factor analysis

To investigate the structural validity of the five-factor structure obtained in the EFA, we conducted a confirmatory factor analysis (CFA) on the CFA sample. The five-factor structure with 25 items showed acceptable fit (Table 3), providing evidence of structural validity (CFI = 0.92; TLI = 0.91; RMSEA = 0.07 [0.06-0.07, 90% CI]). Two equity constraints were imposed on item pairs 32-33 (item 32: “I dim my mobile phone screen within 1 h before attempting to fall asleep.”; item 33: “I dim my computer screen within 1 h before attempting to fall asleep.”) and 16-17 (item 16: “I wear blue-filtering, orange-tinted, and/or red-tinted glasses indoors during the day.”; item 17: “I wear blue-filtering, orange-tinted, and/or red-tinted glasses outdoors during the day.”). Item pair 32-33 describes the preference for dimming the electric devices’ brightness before bedtime, whereas item pair 16-17 represents the use of blue filtering or coloured glasses during the daytime. Given the similar nature of captured behaviours within each item pair, we accepted the imposed equity constraints. Nevertheless, the SRMR value exceeded the guideline recommendation (SRMR = 0.12). In order to improve the model fit, we conducted a post hoc model modification. Firstly, the modification indices suggested cross-loadings between items 37 and 26 (item 37: “I purposely leave a light on in my sleep environment while sleeping.”; item 26: “I turn on my ceiling room light when it is light outside.”), which were hence discarded. Secondly, items 30 and 41 (item 30: “I look at my smartwatch within 1 h before attempting to fall asleep.”; item 41: “I look at my smartwatch when I wake up at night.”) showed a tendency to co-vary in their error variance (MI = 141.127, p < 0.001 ). By allowing the latter pair of items (30 and 41) to co-vary, the model’s error variance attained an improved fit (CFI = 0.95; TLI = 0.95); RMSEA = 0.06 [0.05-0.06, 90% CI]; SRMR = 0.11).

**Figure 4:**
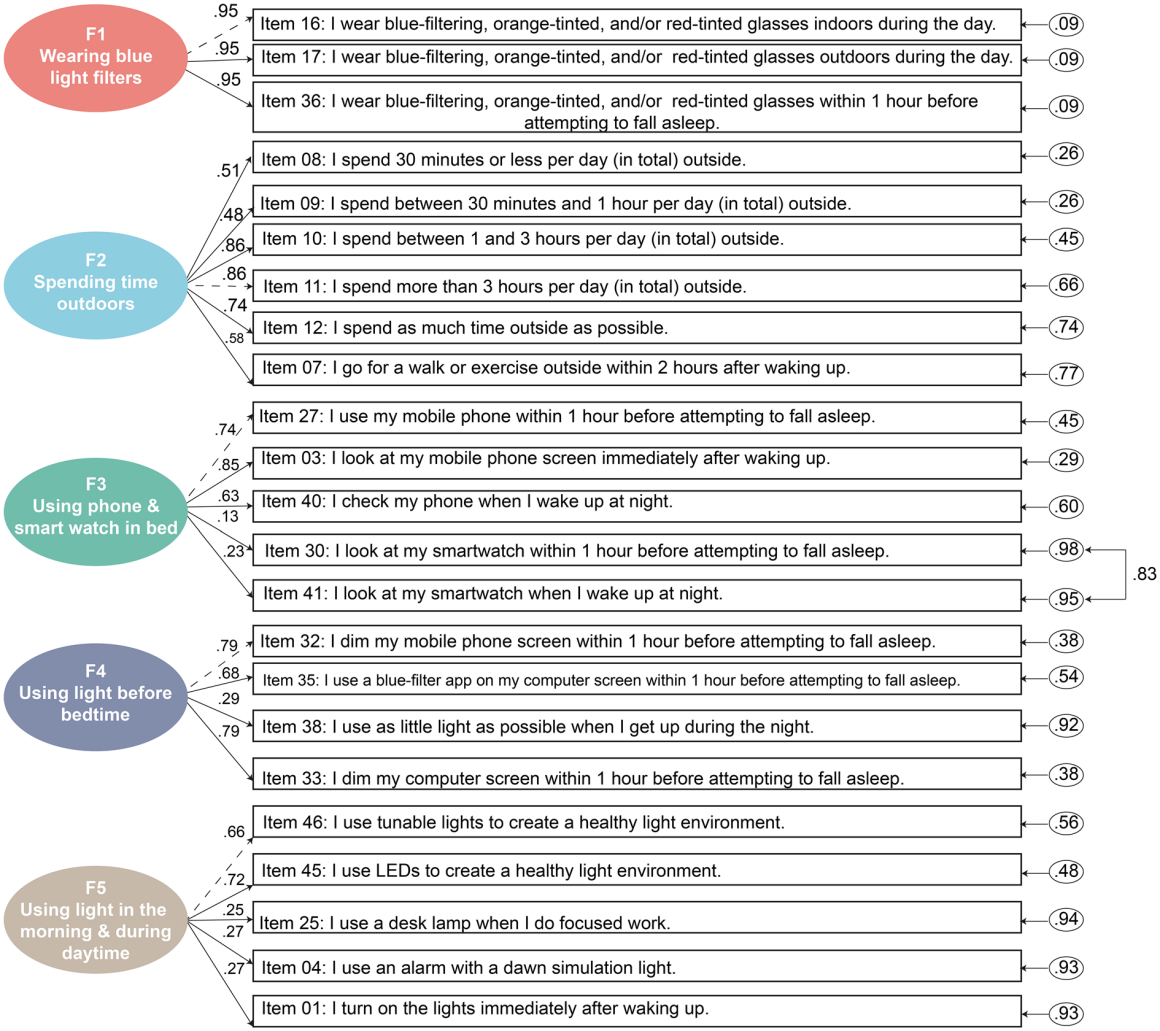
Five-factor model of the LEBA inventory obtained by confirmatory factor analysis. By allowing the error variance of items 41 and 30 to co-vary, our model attained the best fit.

Accordingly, we accept the five-factor model with 23 items, finalizing the long form of the LEBA inventory (see Supplementary File 1). Internal consistency ordinal $\alpha$ values for the five factors of the LEBA were 0.96, 0.83, 0.70, 0.69, and 0.52, respectively. The reliability of the total inventory was satisfactory ($\omega _t$ = 0.68). Figure 4 depicts the obtained CFA structure, while Supplementary Fig. 2 depicts the data distribution and endorsement pattern of the retained 23 items in our CFA sample.

**Table 3:** Confirmatory Factor Analysis model fit indices of the two models: (a) Model 1: five factor model with 25 items (b) Model 2: five factor model with 23 items. Model 2 attained the best fit.

Model	$\chi ^{2}$	df	CFI	TLI	RMSEA	RMSEA 90% Lower CI	RMSEA 90% Upper CI	SRMR
1	675.55	267	0.92	0.91	0.07	0.06	0.07	0.12
2	561.25	231	0.95	0.95	0.07	0.05	0.06	0.11

* df* degrees of freedom, *CFI* comparative fit index, *TLI* Tucker Lewis Index, *RMSEA* root mean square error of approximation, *CI* confidence interval, *SRMR* standardised root mean square.

#### Measurement invariance

We reported the measurement invariance (MI) analysis on the CFA sample based on native (n = 129) and non-native English speakers (n = 133). A detailed demographic description is provided in Supplementary Table 5. Our MI results (Table 4) indicated that LEBA inventory demonstrated the highest level of (residual model) psychometric equivalence across native and non-native English-speaking participants, thus permitting group-mean-based comparisons. The four fitted MI models generated acceptable fit indices, and the model fit did not significantly decrease across the nested models ($\Delta$CFI>-0.01; $\Delta$RMSEA < 0.01).

**Table 4:** Measurement Invariance analysis on CFA sample (n = 262) across native and non-native English speakers.

	$\chi ^{2}$	df	CFI	TLI	RMSEA	RMSEA 90% Lower CI	RMSEA 90% Upper	$\Delta$ $\chi ^{2}$	$\Delta$ df*	p
Configural	632.20	442	0.95	0.94	0.06	0.05	0.07	–	–	–
Metric	643.06	458	0.95	0.95	0.06	0.04	0.07	18.254a	16	0.309
Scalar	711.87	522	0.95	0.95	0.05	0.04	0.06	68.221b	64	0.336
Residual	711.87	522	0.95	0.95	0.05	0.04	0.06	0c	0	NA

* df* degrees of freedom, *CFI* comparative fit index, *TLI* Tucker Lewis Index, *RMSEA* root mean square error of approximation, *CI* confidence interval, *SRMR* standardised root mean square.

a = Metric vs Configural; b = Scalar vs Metric; c = Residual vs Scalar; * = df of model comparison.

### Secondary analysis: Grade level identification and semantic scale network analysis

We investigated the language-based accessibility of the LEBA using Flesch-Kincaid grade-level analysis^31^. Results indicated that at least a language proficiency of educational grade level four (US education system) with age above eight years is required to comprehend the items used in LEBA inventory. Semantic Scale analysis^32^ was administered to assess the LEBA’s (23 items) semantic relation to other questionnaires. The LEBA inventory was most strongly semantically related to scales about sleep: The “Sleep Disturbance Scale For Children”^33^ and the “Composite International Diagnostic Interview (CIDI): Insomnia”^34^. The cosine similarity index ranged between 0.47 and 0.51.

### Developing a short form of the LEBA: IRT-based analysis

Our aim was to provide a data-driven approach to reducing the number of items for cases where a small reduction of items is necessary. In order to derive a short form of the LEBA inventory, we fitted each factor of the LEBA with the graded response model^35^ to the combined EFA and CFA sample (n = 690). The resulting item discrimination parameters of the inventory fell into categories of “very high” (n=10 items), “high” (n=4 items), “moderate” (n=4 items), and “low” (n=5 items), indicating a good range of discrimination along the latent trait level ($\theta$) (Supplementary Table 6). An examination of the item information curve (Supplementary Fig. 3) revealed five items (1, 25, 30, 38, and 41) provided very low information regarding light exposure-related behaviours with relatively flat curves (I($\theta$) < 0.20). We discarded those items, culminating in a short form of the LEBA with five factors and 18 items (Supplementary File 2).

**Figure 5:**
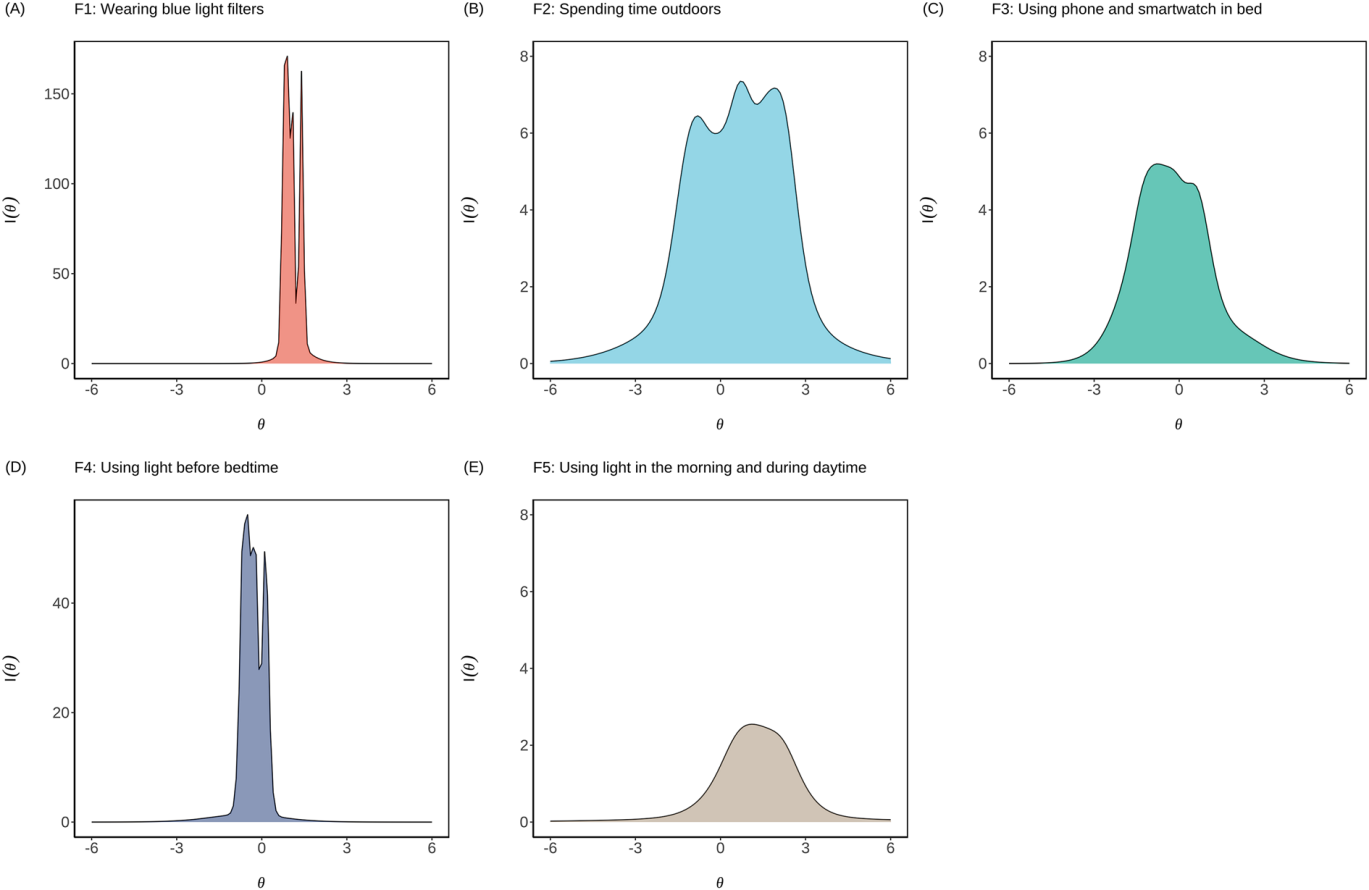
Test information curves for the five factors of the LEBA inventory: (**A**) wearing blue light filters, (**B**) spending time outdoors, (**C**) using a phone and smartwatch in bed, (**D**) using light before bedtime, and (**E**) using light in the morning and during daytime. Along the x-axis, we plotted the underlying latent trait continuum for each factor. Along the y-axis, we plotted how much information a particular factor is carrying across its latent trait continuum.

Subsequently, we obtained five test information curves (TICs). As Fig. 5 illustrates, the TICs of the first and fifth factors peaked on the right side of the centre of their latent traits, while the TICs of the other three factors were roughly centred on the respective trait continuum ($\theta$). This points out that the LEBA short-form estimates the light exposure-related behaviour most precisely near the centre of the trait continuum for the second, third and fourth factors. In contrast, for the first and fifth factors, the TICs were left-skewed, indicating their increased sensitivity in identifying people who are engaging more in those particular light exposure-related behaviour dimensions^36^.

Finally, Supplementary Table 7 summarises the item fit indexes of the LEBA short form. All 18 items yielded an RMSEA value $\le$ 0.06, indicating an adequate fit to the fitted IRT model. Furthermore, Supplementary Fig. 4 depicts the person fit *Zh* statistics histogram for the five IRT models. *Zh* statistics are larger than − 2 for most participants, suggesting a good person fit regarding the selected IRT models.

## Discussion

We have developed two versions of a self-report inventory, the LEBA, that can capture light exposure-related behaviours in multiple dimensions. The 48 generated items were applied in a large-scale, geographically unconstrained, cross-sectional study, yielding 690 completed surveys. To ensure high data quality, participant responses were only included when the five “attention check items” throughout the survey were passed. Ultimately, data was recorded from 74 countries and 28 time zones, including native and non-native English speakers from a sex-balanced and age-diverse sample (see Table 1). The acquired study population complied with our objective to avoid bias from a selective sample, which is crucial when relying on voluntary uncompensated participation.

Data collected in the first round was used to explore the latent structure (EFA sample; n = 428). The exploratory factor analysis revealed a highly interpretable five-factor solution (“Wearing blue light filters”, “Spending time outdoors”, “Using phone and smartwatch in bed”, “Using light before bedtime”, and “Using light in the morning and during daytime”) with 25 items. Our CFA analysis (CFA sample; n = 262) confirmed the five-factor structure we obtained in our EFA, thus providing evidence for structural validity (CFI = 0.95; TLI = 0.95; RMSEA = 0.06). In this model, we discarded two more items (items 26 and 37 ) for possible cross-loadings. As a rule of thumb, reliability coefficients higher than 0.70 are regarded as “satisfactory”. However, at the early developmental stage, a value of 0.50 is considered acceptable^37–39^. Thus, we confer that the internal consistency coefficients ordinal alpha for the five factors and the total inventory were satisfactory (Ordinal alpha ranged between 0.52 to 0.96; McDonald’s $\omega _t$ = 0.68).

The results of the measurement invariance analysis indicate that the construct “light exposure-related behaviour” is equivalent across native and non-native English speakers and, thus, suitable for assessment in both groups. Furthermore, according to the grade level identification method, the LEBA appears understandable for students at least 8.33 years of age visiting grade four or higher. Interestingly, the semantic similarity analysis (“Semantic Scale Network” database^32^) revealed that the “LEBA” is semantically related to the “Sleep Disturbance Scale For Children” (SDSC)^33^ and the “Composite International Diagnostic Interview (CIDI): Insomnia”^34^. Upon inspecting the questionnaire contents, we found that some items in the factors “Using phone and smartwatch in bed” and “Using light before bedtime” have semantic overlap with the SDSC’s and CIDI’s items. However, while the CIDI and the SDSC capture various clinically relevant sleep problems and related activities, the LEBA aims to assess light-exposure-related behaviour. Since light exposure at night has been shown to influence sleep negatively^7,40^, this overlap confirms our aim to measure the physiologically relevant aspects of light-exposure-related behaviour. Nevertheless, the general objectives of the complete questionnaires and the LEBA differ evidently.

While developing and validating LEBA, we have complemented conventional approaches with an Item Response Theory (IRT) analysis. IRT provides a framework to interpret respondents’ obtained scores in the light of latent ability (i.e. light exposure-related behaviour) and the characteristics of the respondents—how they interpret the items^41^. The benefit of implementing IRT analysis was twofold. First, we derived a shorter form of the LEBA inventory (18 items). We fitted a graded response model to the combined EFA and CFA sample (n = 690) and discarded five items (1, 25, 30, 38, and 41) with a relatively flat item information curve [I($\theta$) < 0.20]. The resulting test information curves suggest that the short-LEBA is a psychometrically sound measure with adequate coverage of underlying traits and can be applied to capture the frequency of different light exposure-related behaviours reliably. Often, psychological measurements require the application of several questionnaires simultaneously. Responding to several lengthy questionnaires increases the participants losing focus and becoming tired. Thus, in some circumstances, reducing the number of items even slightly may be necessary to employ the LEBA questionnaire. Our aim was to provide a data-driven approach to reducing the number of items, apart from the possibility of excluding a specific factor from the 23-item questionnaire. Nonetheless, where possible, we strongly recommend using the extended form of the questionnaire to avoid limiting the range of gained information.

The IRT analysis enabled us to capture individual differences in responses to the LEBA items. Findings from the item and person fit index analysis demonstrate that all five fitted models were acceptable and provide evidence of validity for the factors. In addition, the diverse item discrimination parameters indicate an appropriate range of discrimination—the ability to differentiate respondents with different levels of light exposure-related behaviour while acknowledging the interpersonal variability in understanding the item.

### Known limitations

We acknowledge that this work is limited concerning the following aspects:

The fifth factor, “using light in the morning and during daytime”, exhibited low internal consistency both in the exploratory and confirmatory factor analysis (EFA: 0.62; CFA:0.52 ). Since it was above 0.50, considering the developmental phase of this inventory, we accepted the fifth factor. This particular factor captures behaviours related to the usage of light in the morning and daytime. Since light exposure during morning and daytime influences our alertness and cognition^42,43^, we deemed capturing these behaviours essential for the sake of the completeness of our inventory. However, the possibility of improving the reliability should be investigated further by adding more appropriate and relevant items to this factor.

The habitual patterns queried in the developed inventory might not exhaustively represent all relevant light-exposure-related behaviours. For instance, it is conceivable that additional light-related activities not included in the LEBA depend on the respondents’ profession/occupation, geographical context, and socio-economic status. However, we generated the initial item pool with an international team of researchers and followed a thorough psychometric analysis. Therefore, we are confident that the developed LEBA inventory can serve as a good starting point for exploring light exposure-related behaviours in more depth and inform modifications of these behaviours to improve light hygiene.

As with all studies relying on retrospective self-report data, individuals filling in the LEBA may have difficulties precisely recalling the inquired light-related behaviours. In the interest of bypassing a substantial memory component, we limited the recall period to 4 weeks and chose response options that do not require exact memory recall. In contrast to directly assessing light properties via self-report, we assume that reporting behaviours might be more manageable for inexperienced laypeople, as the latter does not rely on existing knowledge about light sources. The comprehensibility of the LEBA is also reflected by the Flesch-Kincaid grade level identification method^31^ which suggested a minimum age of 8.33 years and an educational grade of four or higher (US grading system). We argue that measuring light-related behaviours via self-report is crucial because these behaviours will hardly be as observable by anyone else or measurable with other methods (like behavioural observations) with reasonable effort.

It is important to note that the LEBA utilises a five-point Likert-type response scale which may be susceptible to central tendency bias, i.e. responses are biased towards the central value of the response scale. Future work should evaluate other methods of obtaining responses, such as using a visual-analogue scale.

Finally, there is limited evidence for convergent validity. LEBA, being the first of its kind in characterising light exposure-related *behaviour*, lacks a gold standard at present against which its’ convergent validity evidence could be established. A recent study^44^ demonstrated the predictive validity of LEBA by successfully relating its factors to self-reported chronotype, mood, sleep quality, and cognitive function. The results of their study confirmed that light-related behaviours, as captured by the LEBA, could lead to different light exposure experiences that differentially influence health, wellness and performance. Further work will need to establish the convergent validity of LEBA.

### Future directions

To our knowledge, the LEBA is the first inventory characterising light exposure-related behaviour in a scalable manner. Further evidence for the validity of the LEBA could be obtained by administering it conjointly with objective field measurements of light exposure (e.g. with portable light loggers/wearables), smartphone readouts, as well as subjective data in the form of 24-h recalls. Such a study could explore how (subjectively measured) light exposure-related behavioural patterns translate into (objectively measured) received light exposure, and smartphone use, and how closely the retrospective questionnaire relates to daily reports of these behaviours.

### Conclusion

Here, we developed a novel, internally consistent and structurally valid 23-item self-report inventory for capturing light exposure-related behaviour in five scalable factors. In addition, an 18-item short form of the LEBA was derived using IRT analysis, yielding adequate coverage across the underlying trait continuum. Applying the LEBA inventory can provide insights into light exposure-related habits on a population-based level. Furthermore, it can serve as a good starting point to profile individuals based on their light exposure-related behaviour for health-related interventions.

## Methods

### Data collection

A quantitative cross-sectional, fully anonymous, geographically unconstrained online survey was conducted via REDCap^45,46^ hosted by University of Basel sciCORE. Participants were recruited via the website (https://enlightenyourclock.org/participate-in-research) of the science-communication comic book “Enlighten your clock”, co-released with the survey^47^, social media (i.e., LinkedIn, Twitter, Facebook), mailing lists, word of mouth, the investigators’ personal contacts, and supported by the distribution of the survey link via f.lux^48^. The initial page of the online survey provided information about the study, including that participation was voluntary and that respondents could withdraw from participation at any time without being penalised. Subsequently, consent was recorded digitally for the adult participants (≥18 years), while under-aged participants (<18 years) were prompted to obtain additional assent from their parents/legal guardians. Filling in all questionnaires was estimated to take less than 30 min, and participation was not compensated.

As a part of the demographic data, participants provided information regarding age, sex, gender identity, occupational status, COVID-19-related occupational setting, time zone/country of residence and native language. The demographic characteristics of our sample are given in Table 1. Participants were further asked to confirm that they participated in the survey for the first time. All questions incorporating retrospective recall were aligned to a “past four weeks” period. Additionally, four attention check items were included among the questionnaires to ensure high data quality, with the following phrasing: “We want to make sure you are paying attention. What is 4+5?﻿”; “Please select ‘Strongly disagree’ here.; “Please type in ‘nineteen’ as a number.﻿”; and “Please select ‘Does not apply/I don’t know.’ here﻿.”.

### Analytic strategy

**Figure 6:**
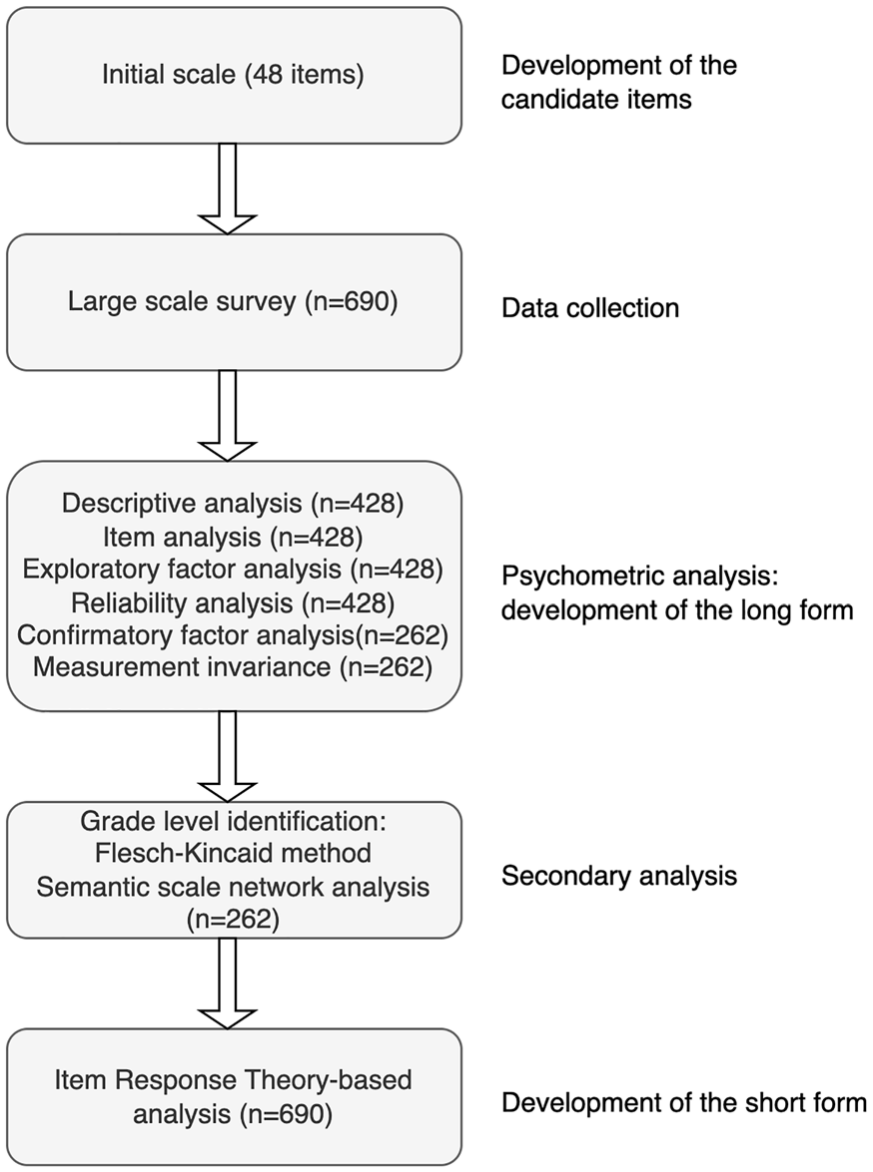
Flow diagram of the development of the LEBA inventory.

Figure 6 summarises the steps we followed while developing the LEBA. We conducted all analyses with the statistical software environment R.

1. We set an item pool of 48 items with a six-point Likert-type response format (0-Does not apply/I don’t know, 1-Never, 2-Rarely, 3-Sometimes, 4-Often, 5-Always) for our initial inventory. Our purpose was to capture light exposure-related behaviour. In that context, the first two response options: “Does not apply/I don’t know” and “Never”, provided similar information. As such, we collapsed them into one, making it a five-point Likert-type response format (1-Never, 2-Rarely, 3-Sometimes, 4-Often, 5-Always).
2. Two rounds of data collection were administered. In the first round (EFA sample; n = 428), we collected data for the exploratory factor analysis (EFA). A sample of at least 250–300 is recommended for EFA^49,50^. The EFA sample exceeded this recommendation. The second round data (CFA sample; n = 262) was subjected to confirmatory factor analysis (CFA). To assess sampling adequacy for CFA, we followed the “N:q” rule^51–54^, where at least ten participants per item are required to earn the trustworthiness of the result. Again, our CFA sample exceeded these guidelines.
3. We conducted descriptive and item analyses and proceeded to EFA on the EFA sample. Prior to the EFA, the necessary assumptions, including sample adequacy, normality assumptions, and quality of correlation matrix, were assessed. As our data violated both the univariate and multivariate normality assumption and yielded ordinal response data, we used a polychoric correlation matrix in the EFA and employed “principal axis” (PA) as the factor extraction method^30,55^. We applied a combination of methods, including a Scree plot^56^, the minimum average partials method^28^, and Hull method^29^ to identify factor numbers. To determine the latent structure, we followed the common guidelines: (i) no factors with fewer than three items, (ii) no factors with a factor loading <0.3, and (iii) no items with cross-loading >0.3 across factors^57^.

Though Cronbach’s internal consistency coefficient alpha is widely used for estimating internal consistency, it tends to deflate the estimates for Likert-type data since the calculation is based on the Pearson correlation matrix, which requires response data to be continuous in nature^58,59^. Subsequently, we reported ordinal alpha for each factor obtained in the EFA, which was suggested as a better reliability estimate for ordinal data^59^. We also estimated the internal consistency reliability of the total inventory using McDonald’s $\omega _t$ coefficient, which was suggested as a better reliability estimate for multidimensional constructs^60,61^. Both ordinal alpha and McDonald’s $\omega _t$ coefficient values range between 0 and 1, where higher values represent better reliability.

To validate the latent structure obtained in the EFA, we conducted a categorical confirmatory factor analysis (CFA) with the weighted least squares means and variance adjusted (WLSMV) estimation^30^ on the CFA sample. We assessed the model fit using standard model fit guidelines: (i) $\chi ^2$ test statistics: a non-significant test statistics is required to accept the model, (ii) comparative fit index (CFI) and Tucker Lewis index (TLI): close to 0.95 or above/ between 0.90-0.95 and above, (iii) root mean square error of approximation (RMSEA): close to 0.06 or below, and (iv) Standardised root mean square (SRMR): close to 0.08 or below^62,63^. However, the $\chi ^2$ test is sensitive to sample size^64^, and SRMR does not work well with ordinal data^65^. Consequently, we judged the model fit using CFI, TLI and RMSEA.

In order to evaluate whether the construct demonstrated psychometric equivalence and the same meaning across native English speakers (n = 129) and non-native English speakers (n = 133) in the CFA sample (n = 262), measurement invariance analysis^53,66^ was used. We used a structural equation modelling framework to assess the measurement invariance. We successively compared four nested models: configural, metric, scalar, and residual models, using the $\chi ^2$ difference test ($\Delta \chi ^2$). Among MI models, the configural model is the least restrictive, and the residual model is the most restrictive. A non-significant $\Delta \chi ^2$ test between two nested measurement invariance models indicates mode fit does not significantly decrease for the superior model, thus allowing the superior invariance model to be accepted^67,68^.

(4) In a secondary analysis, we identified the educational grade level (US education system) required to understand the items in our inventory with the Flesch-Kincaid grade level identification method^31^. Correspondingly, we analyzed possible semantic overlap of our developed inventory using the “Semantic Scale Network” (SSN) engine^32^. The SSN detects semantically related scales and provides a cosine similarity index ranging between − 0.66 and 1^32^. Pairs of scales with a cosine similarity index value of 1 indicate full semantical similarity, suggesting redundancy.
(5) We derived a short form of the LEBA employing an Item Response Theory (IRT) based analysis. We fitted each factor of the LEBA to the combined EFA and CFA sample (n = 690) using the graded response model^35^. IRT assesses the item quality by estimating the item discrimination, item difficulty, item information curve, and test information curve^36^. Item discrimination indicates how well a particular item can differentiate between participants across the given latent trait continuum ($\theta$). Item difficulty corresponds to the latent trait level at which the probability of endorsing a particular response option is 50%. The item information curve (IIC) indicates the amount of information an item carries along the latent trait continuum. Here, we reported the item difficulty and discrimination parameter and categorised the items based on their item discrimination index: (i) none = 0, (ii) very low = 0.01 to 0.34, (iii) low = 0.35 to 0.64, (iv) moderate = 0.65 to 1.34, (v) high = 1.35 to 1.69, (vi) very high >1.70^36^. We discarded the items with a relatively flat item information curve (information <0.2) to derive the short form of the LEBA. We also assessed the precision of the short LEBA utilising the test information curve (TIC). TIC indicates the amount of information a particular scale carries along the latent trait continuum. Additionally, the item and person fit of the fitted IRT models were analyzed to gather more evidence on the validity and meaningfulness of our scale^30^. The item fit was evaluated using the RMSEA value obtained from the Signed-$\chi ^2$ index implementation, where an RMSEA value $\le$0.06 was considered an adequate item fit. The person fit was estimated employing the standardised fit index Zh statistics^69^. Here, *Zh* < − 2 was considered a misfit^69^.

### Ethical approval

The current research project utilises fully anonymous online survey data and, therefore, does not fall under the scope of the Human Research Act, making an authorization from the ethics committee redundant. Nevertheless, the cantonal ethics commission (Ethikkommission Nordwest- und Zentralschweiz, EKNZ) reviewed our proposition (project ID Req-2021-00488) and issued an official clarification of responsibility.

### Supplementary Information

Supplementary Information.

## Data Availability

The present article is a fully reproducible open-access R Markdown document. The reproducibility of this manuscript was confirmed using CODECHECK^70^ (https://codecheck.org.uk), yielding CODECHECK certificate 2023-012 (10.5281/zenodo.10213244). All code and data underlying this article are available on a public GitHub repository (https://github.com/leba-instrument/leba-manuscript). The English version of the long and short forms of the LEBA inventory and online survey implementation templates on common survey platforms (Qualtrics and REDCap) are available on another public GitHub repository (https://github.com/leba-instrument/leba-instrument-en) as well as on the dedicated website of the LEBA inventory (https://leba-instrument.org/) under an open-access licence (Creative Commons CC-BY).
