# Supplementary material for: An inventory of human light exposure behaviour
Source: Sci Rep. 2023 Dec 13;13:22151. doi: 10.1038/s41598-023-48241-y (PMC10719384; doi:10.1038/s41598-023-48241-y)
Supplement: Supplementary file 1 — Supplementary Information. [file 41598_2023_48241_MOESM1_ESM.pdf]

Supplementary File 1

Light Exposure Behaviour Assessment (LEBA): Long Form

Instructions to Participant

Please indicate how often you performed the following behaviours in the **past four weeks**

	**Items**	Never	Rarely	Sometimes	Often	Always
01	I wear blue-filtering, orange-tinted, and/or red-tinted glasses indoors during the day.					
02	I wear blue-filtering, orange-tinted, and/or red-tinted glasses outdoors during the day.					
03	I wear blue-filtering, orange-tinted, and/or red-tinted glasses within 1 hour before attempting to fall asleep.					
04	I spend 30 minutes or less per day (in total) outside. *(Reverse-scored)*					
05	I spend between 30 minutes and 1 hour per day (in total) outside.					
06	I spend between 1 and 3 hours per day (in total) outside.					
07	I spend more than 3 hours per day (in total) outside.					
08	I spend as much time outside as possible.					
09	I go for a walk or exercise outside within 2 hours after waking up.					
10	I use my mobile phone within 1 hour before attempting to fall asleep.					
11	I look at my mobile phone screen immediately after waking up.					
12	I check my phone when I wake up at night.					
13	I look at my smartwatch within 1 hour before attempting to fall asleep.					
14	I look at my smartwatch when I wake up at night.					
15	I dim my mobile phone screen within 1 hour before attempting to fall asleep.					
16	I use a blue-filter app on my computer screen within 1 hour before attempting to fall asleep.					
17	I use as little light as possible when I get up during the night.					
18	I dim my computer screen within 1 hour before attempting to fall asleep.					
19	I use tunable lights to create a healthy light environment.					
20	I use LEDs to create a healthy light environment.					
21	I use a desk lamp when I do focused work.					
22	I use an alarm with a dawn simulation light.					
23	I turn on the lights immediately after waking up.					

Scoring
(Note: R = reverse-scored item)

LEBA captures light exposure-related behaviours on a 5-point Likert-type scale ranging from 1 to 5 (1 = never; 2 = rarely; 3 = sometimes; 4 = often; 5 = always; for reversed-scored item: 1 = always; 2 = often; 3 = sometimes; 4 = rarely; 5 = never). The score of each factor is calculated by using the mean score of the corresponding items.

**Factor Name**	**Score**
F1: Wearing blue light filters	Mean score of items 01, 02, 03
F2: Spending time outdoors	Mean score of items 04(R), 05, 06, 07, 08, 09
F3: Using phone and smartwatch in bed	Mean score of items 10, 11, 12, 13, 14
F4: Using light before bedtime	Mean score of items 15, 16,17, 18
F5: Using light in the morning and during daytime	Mean score of items 19, 20, 21, 22, 23

Supplementary File 2

Light Exposure Behaviour Assessment (LEBA): Short Form

Instructions to Participant

Please indicate how often you performed the following behaviours in the **past four weeks.**

	Items	Never	Rarely	Sometimes	Often	Always
01	I wear blue-filtering, orange-tinted, and/or red-tinted glasses indoors during the day.					
02	I wear blue-filtering, orange-tinted, and/or red-tinted glasses outdoors during the day.					
03	I wear blue-filtering, orange-tinted, and/or red-tinted glasses within 1 hour before attempting to fall asleep.					
04	I spend 30 minutes or less per day (in total) outside. *(Reverse-scored)*					
05	I spend between 30 minutes and 1 hour per day (in total) outside.					
06	I spend between 1 and 3 hours per day (in total) outside.					
07	I spend more than 3 hours per day (in total) outside.					
08	I spend as much time outside as possible.					
09	I go for a walk or exercise outside within 2 hours after waking up.					
10	I use my mobile phone within 1 hour before attempting to fall asleep.					
11	I look at my mobile phone screen immediately after waking up.					
12	I check my phone when I wake up at night.					
15	I dim my mobile phone screen within 1 hour before attempting to fall asleep.					
16	I use a blue-filter app on my computer screen within 1 hour before attempting to fall asleep.					
18	I dim my computer screen within 1 hour before attempting to fall asleep.					
19	I use tunable lights to create a healthy light environment.					
20	I use LEDs to create a healthy light environment.					
22	I use an alarm with a dawn simulation light.					

Scoring

(Note: R = reverse-scored item)

LEBA captures light exposure-related behaviours on a 5-point Likert-type scale ranging from 1 to 5 (1 = never; 2 = rarely; 3 = sometimes; 4 = often; 5 = always; for reversed-scored item: 1 = always; 2 = often; 3 = sometimes; 4 = rarely; 5 = never). The score of each factor is calculated by using the mean score of the corresponding items.

**Factor Name**	**Score**
F1: Wearing blue light filters	Mean score of items 01, 02, 03
F2: Spending time outdoors	Mean score of items 04(R), 05, 06, 07, 08, 09
F3: Using phone and smartwatch in bed	Mean score of items 10, 11, 12
F4: Using light before bedtime	Mean score of items 15, 16, 18
F5: Using light in the morning and during daytime	Mean score of items 19, 20, 22

Supplementary Tables

Supplementary Table 1

List of instruments measuring related constructs to LEBA.

Name	Number of all items	Description	Relevant items	Scale type
Visual Light Sensitivity Questionnaire-8 (Verriotto et al., 2017)	8	To assess the presence and severity of photosensitivity symptoms	None	5-point Likert scale
Office Light Survey (Eklund & Boyce, 1996)	30	To assess electrical lighting environment in office	Item 29	Mixed response format
Harvard Light Exposure Assessment Questionnaire (Bajaj et al., 2011)	1	To assess an individual's daily light exposure	None	Semi-quantitative
Hospital Lighting Survey (Dianat et al., 2013)	23	To assess light environment in a hospital	Items 16, 17	5-point Likert scale
Morningness-Eveningness Questionnaire (Horne & Ostberg, 1976)	19	To assess an individual's chronotype	Items 1, 2, 8, 13, 14	Mixed response format
Munich Chronotype Questionnaire (Roenneberg et al., 2003)	17	To understand an individual's phase of entrainment	Time spent outdoors	Mixed response format
Sleep Practices and Attitudes Questionnaire (Grandner et al., 2014)	16	To assess practice, behaviour and attitude related to sleep	Activities in bed and sleep environment subscales	5-point Likert scale
The Pittsburgh Sleep Quality Index (Buysse et al., 1989)	9	To assess sleep quality and sleeping pattern	items 1-4	Mixed response format
Self-Rating of Biological Rhythm Disorder for Disorder for Adolescents (Xie et al., 2022)	29	To assess four dimensions of biological rhythm disorder in adolescents	Items 3, 6, 22-25, 29	5-point Likert scale
Photosensitivity Assessment Questionnaire (PAQ) (Bossini et al., 2006)	16	To assess “photophobia" and "photophilia"	All items	Binary response option

Supplementary Table 2

Geographical location of the participants (n = 690).

	Time zone and country name	Number of Participants
1	Africa/Ceuta (UTC +01:00)	2
2	Africa/Douala (UTC +01:00)	1
3	Africa/Johannesburg (UTC +02:00)	5
4	Africa/Khartoum (UTC +02:00)	2
5	Africa/Lagos (UTC +01:00)	1
6	America/Adak (UTC -09:00)	2
7	America/Anchorage (UTC -08:00)	3
8	America/Araguaina (UTC -03:00)	2
9	America/Argentina/Buenos_Aires (UTC -03:00)	5
10	America/Argentina/Cordoba (UTC -03:00)	2
11	America/Argentina/Jujuy (UTC -03:00)	1
12	America/Bahia (UTC -03:00)	2
13	America/Blanc-Sablon (UTC -04:00)	1
14	America/Bogota (UTC -05:00)	2
15	America/Boise (UTC -06:00)	4
16	America/Cayman (UTC -05:00)	1
17	America/Chicago (UTC -05:00)	30
18	America/Costa_Rica (UTC -06:00)	2
19	America/Cuiaba (UTC -04:00)	1
20	America/Denver (UTC -06:00)	6
21	America/Detroit (UTC -04:00)	6
22	America/Edmonton (UTC -06:00)	14
23	America/Fortaleza (UTC -03:00)	1
24	America/Guatemala (UTC -06:00)	1
25	America/Guayaquil (UTC -05:00)	2
26	America/Halifax (UTC -03:00)	1
27	America/Indiana/Indianapolis (UTC -04:00)	3
28	America/Indiana/Tell_City (UTC -05:00)	1
29	America/Kentucky/Louisville (UTC -04:00)	3
30	America/Los_Angeles (UTC -07:00)	37
31	America/Martinique (UTC -04:00)	1
32	America/Mexico_City (UTC -06:00)	2
33	America/Moncton (UTC -03:00)	2
34	America/Monterrey (UTC -06:00)	1
35	America/New_York (UTC -04:00)	63
36	America/North_Dakota/Center (UTC -05:00)	1

37	America/North_Dakota/New_Salem (UTC -05:00)	1
38	America/Panama (UTC -05:00)	1
39	America/Phoenix (UTC -07:00)	7
40	America/Resolute (UTC -05:00)	1
41	America/Santiago (UTC -03:00)	8
42	America/Sao_Paulo (UTC -03:00)	19
43	America/Toronto (UTC -04:00)	16
44	America/Vancouver (UTC -07:00)	6
45	Antarctica/Macquarie (UTC +11:00)	1
46	Asia /Taipei City (UTC +08:00)	3
47	Asia/Amman (UTC +03:00)	2
48	Asia/Barnaul (UTC +07:00)	1
49	Asia/Dhaka (UTC +06:00)	1
50	Asia/Famagusta (UTC +02:00)	1
51	Asia/Ho_Chi_Minh (UTC +07:00),British - America/Tortola (UTC -04:00)	2
52	Asia/Hong_Kong (UTC +08:00)	2
53	Asia/Jakarta (UTC +07:00)	9
54	Asia/Jerusalem (UTC +02:00)	4
55	Asia/Karachi (UTC +05:00)	1
56	Asia/Kathmandu (UTC +05:45)	2
57	Asia/Kolkata (UTC +05:30)	38
58	Asia/Kuala_Lumpur (UTC +08:00)	7
59	Asia/Kuching (UTC +08:00)	2
60	Asia/Manila (UTC +08:00)	6
61	Asia/Novosibirsk (UTC +07:00)	1
62	Asia/Riyadh (UTC +03:00)	1
63	Asia/Seoul (UTC +09:00)	1
64	Asia/Shanghai (UTC +08:00)	7
65	Asia/Singapore (UTC +08:00)	1
66	Asia/Tokyo (UTC +09:00)	3
67	Asia/Tomsk (UTC +07:00)	1
68	Asia/Ulaanbaatar (UTC +08:00)	1
69	Asia/Vladivostok (UTC +10:00)	1
70	Asia/Yangon (UTC +06:30)	1
71	Asia/Yekaterinburg (UTC +05:00)	1
72	Atlantic/Canary (UTC)	1
73	Australia/Adelaide (UTC +10:30)	2
74	Australia/Brisbane (UTC +10:00)	4
75	Australia/Darwin (UTC +09:30)	1
76	Australia/Melbourne (UTC +11:00)	5
77	Australia/Perth (UTC +08:00)	2
78	Australia/Sydney (UTC +11:00)	2

79	East Africa/Dodoma (UTC +03:00)	1
80	Europe/Amsterdam (UTC +01:00)	19
81	Europe/Athens (UTC +02:00)	3
82	Europe/Belgrade (UTC +01:00)	3
83	Europe/Berlin (UTC +01:00)	53
84	Europe/Bratislava (UTC +01:00)	2
85	Europe/Brussels (UTC +01:00)	4
86	Europe/Bucharest (UTC +02:00)	3
87	Europe/Budapest (UTC +01:00)	2
88	Europe/Busingen (UTC +01:00)	3
89	Europe/Copenhagen (UTC +01:00)	3
90	Europe/Dublin (UTC)	5
91	Europe/Helsinki (UTC +02:00)	9
92	Europe/Istanbul (UTC +03:00)	6
93	Europe/Kiev (UTC +02:00)	1
94	Europe/Lisbon (UTC)	2
95	Europe/Ljubljana (UTC +01:00)	3
96	Europe/London (UTC)	57
97	Europe/Madrid (UTC +01:00)	7
98	Europe/Moscow (UTC +03:00)	8
99	Europe/Oslo (UTC +01:00)	3
100	Europe/Paris (UTC +01:00)	22
101	Europe/Prague (UTC +01:00)	3
102	Europe/Riga (UTC +02:00)	2
103	Europe/Rome (UTC +01:00)	9
104	Europe/Sofia (UTC +02:00)	1
105	Europe/Stockholm (UTC +01:00)	4
106	Europe/Tallinn (UTC +02:00)	2
107	Europe/Tirane (UTC +01:00)	1
108	Europe/Vienna (UTC +01:00)	1
109	Europe/Vilnius (UTC +02:00)	5
110	Europe/Warsaw (UTC +01:00)	15
111	Europe/Zagreb (UTC +01:00)	2
112	Europe/Zurich (UTC +01:00)	21
113	European /Skopje (UTC +01:00)	1
114	Iran /Tehran (UTC +0:30)	3
115	Pacific/Auckland (UTC +13:00)	6
116	Pacific/Chatham (UTC +13:45)	1
117	Pacific/Easter (UTC -05:00)	1
118	Pacific/Honolulu (UTC -10:00)	2

Supplementary Table 3.

Minimum average partial (MAP) method of factor number determination. MAP Statistic is the lowest in the 5th row, indicating five factors are required.

MAP Statistic[a]	df[b]	χ^2	RMSEA[c]	BIC[d]	SRMR[e]
0.01125	1080	4344.31	0.08	-2199.54	0.09
0.01062	1033	3735.35	0.08	-2523.72	0.08
0.01077	987	3065.44	0.07	-2914.91	0.07
0.01042	942	2661.78	0.07	-3045.92	0.06
0.0093	898	2237.56	0.06	-3203.53	0.06
0.0094	855	2040.02	0.06	-3140.53	0.05
0.0097	813	1861.69	0.05	-3064.37	0.04
0.0100	772	1620.64	0.05	-3057.00	0.04

Note. [a] Minimum average partial; [b] Degrees of Freedom; [c] Root Mean Square Error of Approximation; [d] Schwarz's Bayesian Information Criterion; [e] Standardized Root Mean Square.

Supplementary Table 4

Factor loadings and communality of the retained in EFA with six factors. One factor emerged with only two items (n = 428).

Items	PA1	PA2	PA3	PA4	PA5	PA6	Communality
Item 16	.99						.01
Item 36	.94						.10
Item 17	.80						.33
Item 11		.82					.30
Item 10		.81					.34
Item 12		.64					.53
Item 08		-.48					.75
Item 07		.47					.74
Item 09		.33					.88
Item 33			.97				.02
Item 32			.77				.31
Item 35			.54				.59
Item 31			.49				.67
Item 03				.84			.27
Item 27				.81			.33
Item 40				.69			.47
Item 46					.65		.48
Item 45					.57		.65
Item 04					.48		.67
Item 25					.40		.76
Item 01					.35		.87
Item 26					.35		.84
Item 37						-.8	.32
Item38						.39	.76
% Of Variance	11	10	9	9	6	5	-

Note. Only loadings higher than.30 are reported.

Supplementary Table 5

Demographics Characteristics of the native and non-native English Speakers (n = 262).

Variable	Overall [1] (n = 262)	Native English Speakers[1] (n = 129)	Non-native English Speakers[1] (n = 133)
**Age**	32.89 (13.66)	34.08 (15.32)	31.74 (11.77)
**Sex**			
Female	136 (52%)	80 (62%)	56 (42%)
Male	121 (46%)	48 (37%)	73 (55%)
Other	5 (1.9%)	1 (.08%)	4 (3.0%)
**Occupational Status**			
Work	161 (61%)	76 (59%)	85 (64%)
School	52 (20%)	27 (21%)	25 (19%)
Neither	49 (19%)	26 (20%)	23 (17%)
**Occupational Setting**			
Home Office/Home schooling	109 (42%)	50 (39%)	59 (44%)
Face-to-face work/Face-to-face schooling	41 (16%)	22 (17%)	19 (14%)
Combination of home and face-to-face work/schooling	53 (20%)	23 (18%)	30 (23%)
Neither (no work or school, or in vacation)	59 (23%)	34 (26%)	25 (19%)

[1] Mean (SD); n (%).

Supplementary Table 6

Items discrimination and response category difficulty thresholds of 23 items in LEBA (n = 690).

Items	a	b_1	b_2	b_3	b_4	Item Discrimination Category
**F1: Wearing blue light filters**						
Item 16	28.13	0.78	0.90	1.06	1.40	Very High
Item 36	4.49	0.94	1.08	1.23	1.40	Very High
Item 17	2.81	0.97	1.11	1.38	1.62	Very High
**F2: Spending time outdoors**						
Item 11	3.27	-0.79	0.65	1.54	2.31	Very High
Item 10	3.07	-1.27	-0.09	0.82	2.00	Very High
Item 12	1.72	-0.67	0.44	1.28	2.11	Very High
Item 07	1.09	-0.50	0.73	1.63	2.97	Moderate
Item 08	1.19	-2.26	-0.48	0.64	1.91	Moderate
Item 09	0.91	-2.63	-0.96	1.11	3.49	Moderate
**F3: Using phone and smartwatch in bed**						
Item 27	2.21	-1.88	-1.19	-0.73	0.30	Very High
Item 03	3.03	-1.24	-0.77	-0.20	0.66	Very High
Item 40	1.55	-0.51	0.46	1.32	2.22	High
Item 30	0.49	3.27	3.74	4.64	6.52	Low
Item 41	0.51	3.87	4.78	6.39	8.91	Low
**F4: Using light before bedtime**						
Item 32	1.62	-1.03	-0.78	-0.42	0.16	High
Item 35	1.37	-1.09	-0.98	-0.75	-0.40	High
Item 38	0.40	-7.48	-5.56	-4.23	-0.90	Low
Item 33	12.31	-0.66	-0.48	-0.24	0.13	Very High
**F5: Using light in the morning and during daytime**						
Item 46	2.22	0.68	0.89	1.38	2.17	Very High
Item 45	1.51	0.30	0.55	1.17	1.91	High
Item 25	0.52	-1.37	-0.04	1.89	4.22	Low
Item 04	0.84	2.44	2.80	3.18	3.67	Moderate
Item 01	0.39	-0.91	1.52	3.25	5.53	Low

Note. a = item discrimination parameter; b(1-4) = response category difficulty parameter

Supplementary Table 7

Item discrimination, response category difficulty thresholds and fit statistics of the 18 items in short LEBA (n = 690).

Items	a	b_1	b_2	b_3	b_4	Signed χ^2	df	RMSEA	p	Item Discrimination Category
**F1: Wearing blue light filters**										
Item 16	28.13	0.78	0.90	1.06	1.40	2.02	6	0.00	0.92	Very High
Item 36	4.49	0.94	1.08	1.23	1.40	39.07	13	0.05	0.00	Very High
Item 17	2.81	0.97	1.11	1.38	1.62	25.58	13	0.04	0.02	Very High
**F2: Spending time outdoors**										
Item 11	3.27	-0.79	0.65	1.54	2.31	55.03	27	0.04	0.00	Very High
Item 10	3.07	-1.27	-0.09	0.82	2.00	53.19	30	0.03	0.01	Very High
Item 12	1.72	-0.67	0.44	1.28	2.11	34.39	42	0.00	0.79	Very High
Item 07	1.09	-0.50	0.73	1.63	2.97	67.45	46	0.03	0.02	Moderate
Item 08	1.19	-2.26	-0.48	0.64	1.91	140.90	46	0.05	0.00	Moderate
Item 09	0.91	-2.63	-0.96	1.11	3.49	131.19	45	0.05	0.00	Moderate
**F3: Using phone and smartwatch in bed**										
Item 27	2.12	-1.91	-1.21	-0.74	0.31	16.41	11	0.03	0.13	Very High
Item 03	3.24	-1.22	-0.76	-0.20	0.65	15.09	11	0.02	0.18	Very High
Item 40	1.57	-0.50	0.45	1.30	2.20	9.92	9	0.01	0.36	High
**F4: Using light before bedtime**										
Item 32	1.60	-1.04	-0.79	-0.42	0.16	41.33	15	0.05	0.00	High
Item 35	1.34	-1.10	-0.99	-0.76	-0.41	41.71	14	0.05	0.00	High
Item 33	15.66	-0.66	-0.48	-0.24	0.13	46.89	14	0.06	0.00	Very High
**F5: Using light in the morning and during daytime**										
Item 46	2.34	0.66	0.88	1.36	2.12	19.00	15	0.02	0.21	Very High
Item 45	1.51	0.30	0.55	1.17	1.91	15.05	15	0.00	0.45	High
Item 25	0.49	-1.45	-0.04	1.99	4.46	31.60	15	0.04	0.01	Low

Note. a = item discrimination parameter; $b_{(1-4)}$ = response category difficulty parameter

References (Supplementary Materials)

Bajaj, A., Rosner, B., Lockley, S. W., & Schernhammer, E. S. (2011). Validation of a Light Questionnaire with Real-life Photopic Illuminance Measurements: the Harvard Light Exposure Assessment Questionnaire. *Cancer Epidemiology Biomarkers & Prevention*, *20*(7), 1341-1349. https://doi.org/10.1158/1055-9965.epi-11-0204

Bossini, L., Valdagno, M., Padula, L., De Capua, A., Pacchierotti, C., & Castrogiovanni, P. (2006). Sensibilità alla luce e psicopatologia: Validazione del Questionario per la Valutazione della Fotosensibilità (QVF). *Med Psicosomatica*, *51*, 167-176.

Buysse, D. J., Reynolds, C. F., 3rd, Monk, T. H., Berman, S. R., & Kupfer, D. J. (1989). The Pittsburgh Sleep Quality Index: a new instrument for psychiatric practice and research. *Psychiatry Res*, *28*(2), 193-213. https://doi.org/10.1016/0165-1781(89)90047-4

Dianat, I., Sedghi, A., Bagherzade, J., Asghari Jafarabadi, M., & Stedmon, A. (2013). Objective and subjective assessments of lighting in a hospital setting: Implications for health, safety and performance. *Ergonomics*, *56*. https://doi.org/10.1080/00140139.2013.820845

Eklund, N. H., & Boyce, P. R. (1996). The Development of a Reliable, Valid, and Simple Office Lighting Survey. *Journal of the Illuminating Engineering Society*, *25*(2), 25-40. https://doi.org/10.1080/00994480.1996.10748145

Grandner, M. A., Jackson, N., Gooneratne, N. S., & Patel, N. P. (2014). The development of a questionnaire to assess sleep-related practices, beliefs, and attitudes. *Behavioral sleep medicine*, *12*(2), 123-142. https://doi.org/10.1080/15402002.2013.764530

Horne, J. A., & Ostberg, O. (1976). A self-assessment questionnaire to determine morningness-eveningness in human circadian rhythms. *Int J Chronobiol*, *4*(2), 97-110.

Roenneberg, T., Wirz-Justice, A., & Merrow, M. (2003). Life between Clocks: Daily Temporal Patterns of Human Chronotypes. *Journal of Biological Rhythms*, *18*(1), 80-90. https://doi.org/10.1177/0748730402239679

Verriotto, J. D., Gonzalez, A., Aguilar, M. C., Parel, J.-M. A., Feuer, W. J., Smith, A. R., & Lam, B. L. (2017). New methods for quantification of visual photosensitivity threshold and symptoms. *Translational vision science & technology*, *6*(4), 18-18.

Xie, Y., Wu, X., Tao, S., Wan, Y., & Tao, F. (2022). Development and validation of the self-rating of biological rhythm disorder for Chinese adolescents. *Chronobiol Int*, *39*(2), 198-204. https://doi.org/10.1080/07420528.2021.1989450

Supplementary Figures

Light Exposure Behavior Assessment
Summary Descriptives EFA Sample (n =428)

Items	Summary Statistics						Graphics		Response Pattern				
Item	Mean	SD	Skew	Kurtosis	SW†	Item Total Correlation	Histogram	Density	Never	Rarely	Sometimes	Often	Always
item01	2.27	1.39	0.74	-0.81	0.81*	0.19			42.29% (181)	22.20% (95)	12.62% (54)	12.38% (53)	10.51% (45)
item02	2.87	1.59	0.08	-1.60	0.83*	0.28			31.78% (136)	15.65% (67)	9.35% (40)	20.09% (86)	23.13% (99)
item03	3.36	1.38	-0.48	-1.03	0.87*	0.23			15.89% (68)	11.45% (49)	17.29% (74)	31.07% (133)	24.30% (104)
item04	1.47	1.18	2.38	4.00	0.43*	0.24			84.11% (360)	3.50% (15)	2.10% (9)	2.10% (9)	8.18% (35)
item05	4.01	1.40	-1.22	0.07	0.70*	0.17			12.85% (55)	3.50% (15)	9.58% (41)	17.52% (75)	56.54% (242)
item06	2.79	1.55	0.19	-1.48	0.85*	0.13			32.01% (137)	15.42% (66)	15.89% (68)	15.42% (66)	21.26% (91)
item07	2.26	1.25	0.70	-0.60	0.85*	0.32			35.98% (154)	27.80% (119)	17.29% (74)	12.38% (53)	6.54% (28)
item08	2.97	1.20	-0.06	-0.94	0.91*	0.25			13.79% (59)	22.20% (95)	27.80% (119)	25.93% (111)	10.28% (44)
item09	2.94	1.03	-0.12	-0.40	0.91*	0.08			10.28% (44)	19.63% (84)	41.82% (179)	22.43% (96)	5.84% (25)
item10	2.74	1.04	0.09	-0.74	0.91*	0.42			11.92% (51)	31.31% (134)	31.31% (134)	21.96% (94)	3.50% (15)
item11	2.18	0.90	0.60	0.12	0.86*	0.41			22.43% (96)	46.26% (198)	23.13% (99)	7.01% (30)	1.17% (5)
item12	2.36	1.22	0.59	-0.62	0.87*	0.48			29.91% (128)	29.67% (127)	21.50% (92)	12.15% (52)	6.78% (29)
item13	2.73	1.46	0.20	-1.36	0.87*	0.25			30.14% (129)	17.52% (75)	17.76% (76)	18.69% (80)	15.89% (68)
item14	2.14	1.31	0.77	-0.78	0.80*	0.28			47.20% (202)	18.93% (81)	12.62% (54)	15.65% (67)	5.61% (24)
item15	3.26	1.09	-0.26	-0.45	0.91*	0.03			7.48% (32)	13.79% (59)	37.15% (159)	28.04% (120)	13.55% (58)
item16	1.56	1.23	2.00	2.45	0.50*	0.28			79.67% (341)	4.21% (18)	3.97% (17)	4.67% (20)	7.48% (32)
item17	1.54	1.21	2.07	2.75	0.49*	0.21			80.61% (345)	3.27% (14)	5.14% (22)	3.27% (14)	7.71% (33)
item18	1.12	0.49	5.02	27.80	0.25*	0.18			93.22% (399)	3.50% (15)	2.10% (9)	0.70% (3)	0.47% (2)
item19	1.05	0.36	7.23	52.98	0.13*	0.17			97.43% (417)	0.93% (4)	0.47% (2)	1.17% (5)	0.00% (0)
item20	1.04	0.33	8.99	85.28	0.10*	0.16			98.36% (421)	0.23% (1)	0.70% (3)	0.47% (2)	0.23% (1)
item21	1.14	0.59	4.79	24.05	0.25*	0.21			93.69% (401)	1.64% (7)	3.04% (13)	0.47% (2)	1.17% (5)
item22	3.57	1.07	-0.65	-0.17	0.88*	0.20			4.91% (21)	11.92% (51)	21.96% (94)	43.22% (185)	17.99% (77)
item23	2.56	1.27	0.33	-1.00	0.89*	0.08			26.40% (113)	25.23% (108)	22.66% (97)	17.76% (76)	7.94% (34)
item24	4.14	0.99	-1.23	1.14	0.79*	0.22			2.34% (10)	5.84% (25)	10.98% (47)	37.38% (160)	43.46% (186)
item25	2.59	1.41	0.27	-1.27	0.86*	0.15			34.35% (147)	13.79% (59)	22.20% (95)	17.99% (77)	11.68% (50)
item26	2.25	1.27	0.69	-0.64	0.84*	0.08			38.32% (164)	23.36% (100)	20.09% (86)	10.98% (47)	7.24% (31)
item27	3.80	1.29	-0.87	-0.42	0.82*	0.17			8.41% (36)	11.21% (48)	11.21% (48)	30.37% (130)	38.79% (166)
item28	3.76	1.14	-0.68	-0.45	0.86*	0.18			3.97% (17)	13.08% (56)	17.06% (73)	34.81% (149)	31.07% (133)
item29	2.44	1.31	0.38	-1.14	0.86*	0.13			34.35% (147)	20.33% (87)	19.39% (83)	19.16% (82)	6.78% (29)
item30	1.48	1.11	2.18	3.35	0.48*	0.13			81.78% (350)	3.27% (14)	4.91% (21)	5.37% (23)	4.67% (20)
item31	3.00	1.62	-0.08	-1.61	0.83*	0.39			31.31% (134)	10.05% (43)	11.68% (50)	20.79% (89)	26.17% (112)
item32	3.55	1.65	-0.60	-1.34	0.76*	0.33			23.13% (99)	7.01% (30)	8.18% (35)	14.95% (64)	46.73% (200)
item33	3.62	1.64	-0.68	-1.25	0.74*	0.37			21.96% (94)	7.01% (30)	7.24% (31)	14.49% (62)	49.30% (211)
item34	3.42	1.83	-0.45	-1.69	0.69*	0.20			33.64% (144)	3.04% (13)	3.04% (13)	8.64% (37)	51.64% (221)
item35	3.86	1.67	-0.99	-0.85	0.65*	0.20			22.90% (98)	1.87% (8)	3.74% (16)	9.35% (40)	62.15% (266)
item36	1.54	1.25	2.13	2.86	0.46*	0.35			82.24% (352)	3.04% (13)	3.04% (13)	2.34% (10)	9.35% (40)
item37	1.33	0.91	3.03	8.43	0.41*	0.09			84.58% (362)	7.01% (30)	3.04% (13)	1.64% (7)	3.74% (16)
item38	4.30	1.08	-1.79	2.53	0.67*	0.32			5.37% (23)	3.50% (15)	5.37% (23)	27.57% (118)	58.18% (249)
item39	1.96	0.98	1.02	0.69	0.82*	0.07			37.62% (161)	38.79% (166)	15.65% (67)	5.61% (24)	2.34% (10)
item40	2.16	1.19	0.71	-0.54	0.84*	0.25			39.49% (169)	25.00% (107)	19.63% (84)	11.45% (49)	4.44% (19)
item41	1.31	0.81	2.75	6.92	0.43*	0.14			85.05% (364)	4.67% (20)	6.07% (26)	3.04% (13)	1.17% (5)
item42	3.93	1.48	-1.06	-0.44	0.71*	0.15			14.72% (63)	5.84% (25)	7.94% (34)	14.95% (64)	56.54% (242)
item43	1.64	1.18	1.79	2.02	0.60*	0.22			71.26% (305)	9.35% (40)	10.05% (43)	2.80% (12)	6.54% (28)
item44	3.51	1.30	-0.70	-0.59	0.85*	0.40			13.55% (58)	7.24% (31)	18.69% (80)	35.98% (154)	24.53% (105)
item45	2.22	1.48	0.71	-1.02	0.76*	0.29			53.04% (227)	7.01% (30)	16.36% (70)	11.92% (51)	11.68% (50)
item46	1.76	1.23	1.35	0.44	0.66*	0.39			67.06% (287)	7.71% (33)	11.68% (50)	8.88% (38)	4.67% (20)
item47	2.11	1.17	0.77	-0.39	0.83*	0.37			41.12% (176)	24.77% (106)	20.09% (86)	9.81% (42)	4.21% (18)
item48	2.60	1.25	0.29	-0.86	0.89*	0.36			25.00% (107)	21.50% (92)	30.84% (132)	13.79% (59)	8.88% (38)

† Shapiro–Wilk test

Supplementary Figure 1. Summary descriptive statistics and response pattern of EFA sample (n = 428). '*' denotes a significant deviation from the normality assumption according to the Shapiro-Wilk test. All items violated normality assumptions.

Light Exposure Behavior Assessment

Summary Descriptives CFA Sample (n=262)

Items	Summary Statistics				Graphics		Response Pattern				
	n	Mean	Median	SD	Histogram	Density	Never	Rarely	Sometimes	Often	Always
**F1: Wearing blue light filters**											
item16	262	1.6	1.0	1.3			78.24% (205)	3.44% (9)	4.20% (11)	5.73% (15)	8.40% (22)
item17	262	1.6	1.0	1.2			80.15% (210)	3.44% (9)	5.34% (14)	2.67% (7)	8.40% (22)
item36	262	1.6	1.0	1.3			80.53% (211)	3.44% (9)	3.05% (8)	3.44% (9)	9.54% (25)
**F2: Spending time outdoors**											
item07	262	2.1	2.0	1.2			43.13% (113)	23.66% (62)	14.50% (38)	14.12% (37)	4.58% (12)
item08	262	3.0	3.0	1.2			14.12% (37)	22.90% (60)	20.99% (55)	32.06% (84)	9.92% (26)
item09	262	2.9	3.0	1.1			12.98% (34)	22.14% (58)	34.35% (90)	26.34% (69)	4.20% (11)
item10	262	2.6	3.0	1.1			17.56% (46)	29.39% (77)	29.01% (76)	21.37% (56)	2.67% (7)
item11	262	2.1	2.0	0.9			25.95% (68)	46.56% (122)	20.23% (53)	5.34% (14)	1.91% (5)
item12	262	2.3	2.0	1.2			32.06% (84)	30.92% (81)	19.08% (50)	11.45% (30)	6.49% (17)
**F3: Using phone and smart-watch in bed**											
item03	262	3.7	4.0	1.3			11.83% (31)	7.25% (19)	17.56% (46)	28.24% (74)	35.11% (92)
item27	262	4.0	4.0	1.2			6.11% (16)	7.25% (19)	8.02% (21)	33.59% (88)	45.04% (118)
item30	262	1.4	1.0	1.1			83.59% (219)	2.67% (7)	4.20% (11)	6.11% (16)	3.44% (9)
item40	262	2.5	2.0	1.3			30.92% (81)	27.10% (71)	18.70% (49)	12.21% (32)	11.07% (29)
item41	262	1.2	1.0	0.7			90.08% (236)	3.82% (10)	2.29% (6)	2.67% (7)	1.15% (3)
**F4: Using light before bedtime**											
item32	262	3.4	4.0	1.7			25.95% (68)	4.20% (11)	11.45% (30)	16.79% (44)	41.60% (109)
item33	262	3.1	3.0	1.7			32.44% (85)	6.11% (16)	11.83% (31)	14.12% (37)	35.50% (93)
item35	262	3.6	5.0	1.8			27.48% (72)	2.67% (7)	7.25% (19)	6.49% (17)	56.11% (147)
item38	262	4.3	5.0	1.1			4.20% (11)	7.63% (20)	6.49% (17)	21.37% (56)	60.31% (158)
**F5: Using light in the morning and during daytime**											
item01	262	2.3	2.0	1.4			40.46% (106)	22.52% (59)	14.50% (38)	10.69% (28)	11.83% (31)
item04	262	1.3	1.0	0.8			89.31% (234)	2.29% (6)	3.44% (9)	3.05% (8)	1.91% (5)
item25	262	2.5	2.0	1.4			32.82% (86)	18.32% (48)	21.76% (57)	16.79% (44)	10.31% (27)
item45	262	2.0	1.0	1.4			64.12% (168)	5.34% (14)	9.54% (25)	11.83% (31)	9.16% (24)
item46	262	1.6	1.0	1.2			75.57% (198)	2.67% (7)	8.02% (21)	9.54% (25)	4.20% (11)

Supplementary Figure 2. Summary descriptive statistics and response patterns of the CFA sample (n = 262).

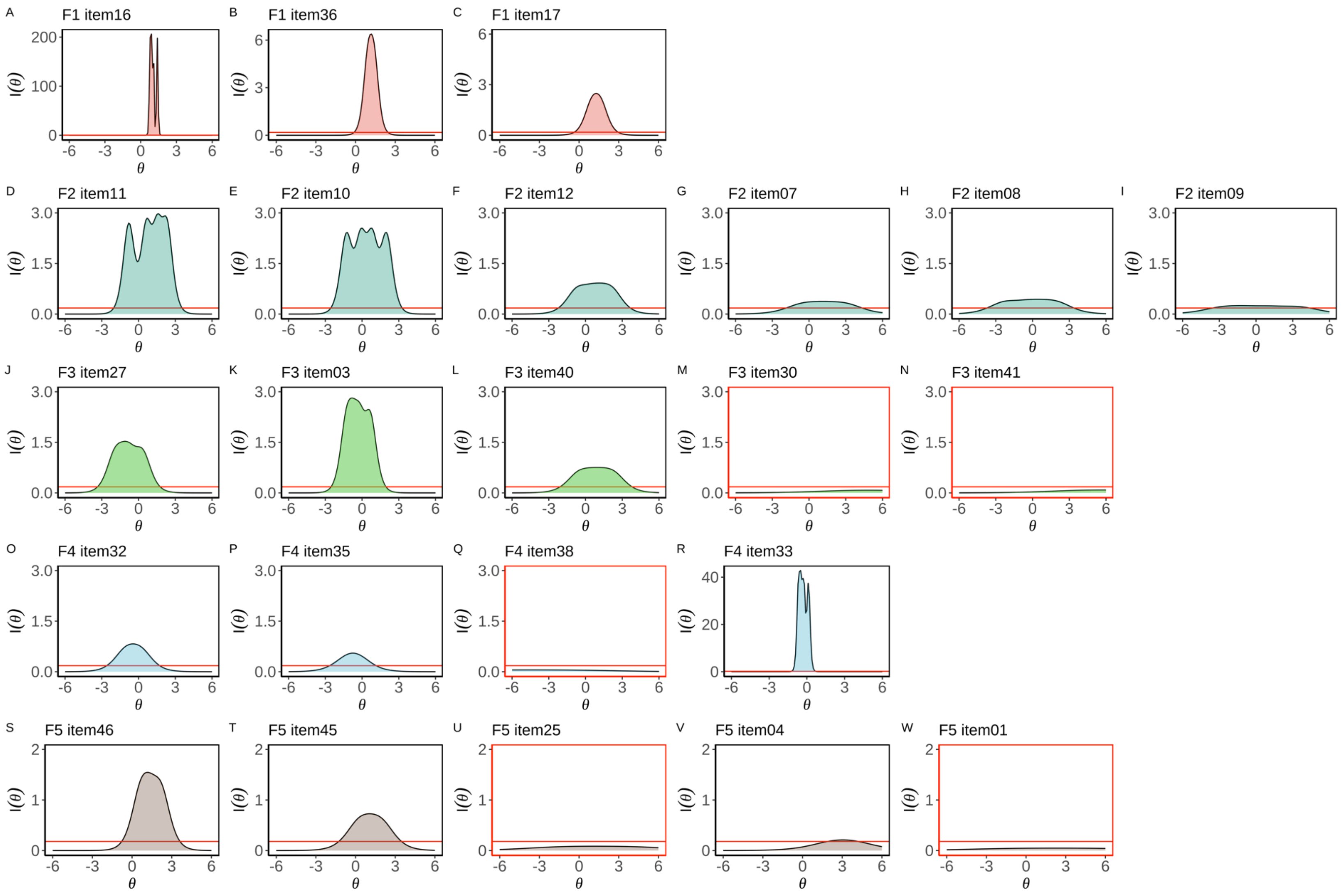

Supplementary Figure 3. Item information curves for all items of LEBA. The five items in red boxes (1, 25, 30, 38, 41) had relatively flat information curves.

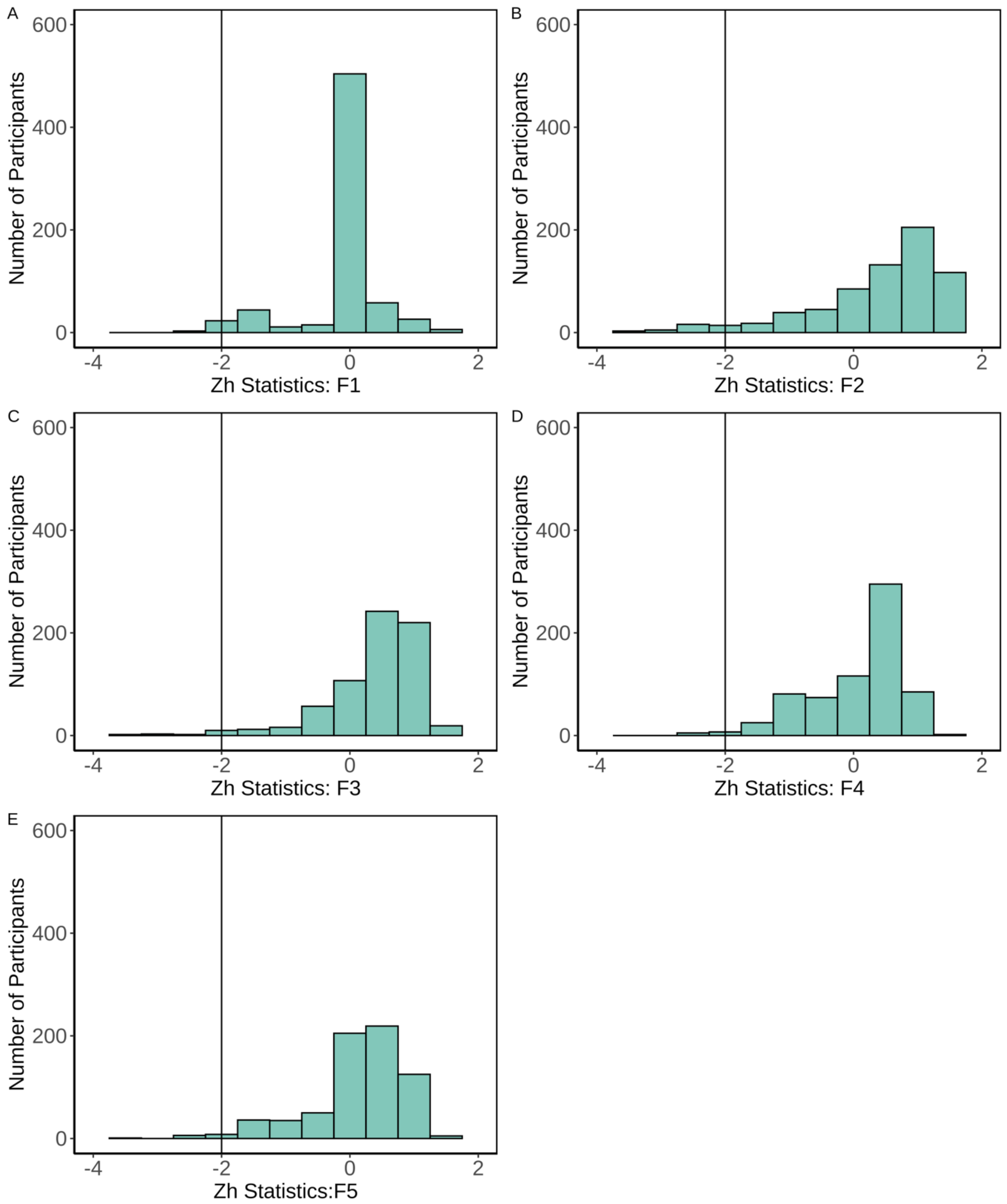

Supplementary Figure 4. Person fit of the five fitted IRT models (a) wearing blue light filters, (b) spending time outdoors, (c) using phone and smart watch in bed, (d) using light before bedtime, and (e) using light in the morning and during daytime. Most of the Zh values are higher than -2.
